# Nonlethal predator effects on the turn-over of wild bird flocks

**DOI:** 10.1038/srep33476

**Published:** 2016-09-16

**Authors:** Bernhard Voelkl, Josh A. Firth, Ben C. Sheldon

**Affiliations:** 1 Edward Grey Institute, Department of Zoology, University of Oxford, South Parks Road, OX1 3PS Oxford, United Kingdom; 2 Animal Welfare Division, University of Bern, Laenggassstrasse 120, 3012 Bern, Switzerland

## Abstract

Nonlethal predator effects arise when individuals of a prey species adjust their behaviour due to the presence of predators. Non-lethal predator effects have been shown to affect social group structure and social behaviour as well as individual fitness of the prey. In this experimental study, we used model sparrowhawks to launch attacks on flocks of wild great tits and blue tits whilst monitoring their social dynamics. We show that nonlethal attacks caused instantaneous turn-over and mixing of group composition within foraging flocks. A single experimental ‘attack’ lasting on average less than three seconds, caused the amount of turn-over expected over three hours (2.0–3.8 hours) of undisturbed foraging. This suggests that nonlethal predator effects can greatly alter group composition within populations, with potential implications for social behaviour by increasing the number of potential interaction partners, as well as longer-term consequences for pair formation and emergent effects determined by social structure such as information and disease transmission. We provide the first evidence, to our knowledge, based on in depth monitoring of a social network to comprehensively support the hypothesis that predators influence the social structure of groups, which offers new perspectives on the key drivers of social behaviour in wild populations.

Predation has long been argued to be a major selective force in evolution, affecting numerous biological properties, including mating systems, socio-spatial structure, group organization and social behaviour^1^,^2^,^3^,^4^. However, most attention in the study of predation effects has been given to lethal effects of predation, while nonlethal effects through the mere presence of predators have received much less attention^5^,^6^,^7^. Nonlethal effects, also sometimes referred to as ‘risk effects’^8^, arise when prey alter their behaviour in response to the presence of predators^5^,^9^. Such effects have been most clearly demonstrated through substantial changes in prey density and population demography caused by predators that were experimentally rendered nonlethal. For example, Peckarsky and collaborators^8^ ‘disarmed’ predatory stone flies (*Megarcys* sp. and *Kogotus* sp.) by gluing together their mouthparts. They then compared the demography of predator free populations of mayflies (*Baetis bicaudatus*) with those of mayfly populations with either nonlethal predators or un-treated, and hence lethal, predators being present. Nonlethal predator effects have been reported for a variety of species and meta-analyses of the existing literature suggest that the impact of ‘intimidation’ by predators on prey demographics can be even considerably stronger than direct consumption^7^,^9^,^10^.

The most common behavioural responses to predation risk include changes in habitat use^11^,^12^, movement patterns^13^,^14^,^15^, vigilance^16^,^17^, escape distance^18^, and foraging behaviour^19^,^20^, which can lead to reduced energy income, lower mating success and increased vulnerability to other predators^10^. The formation of groups can be an effective anti-predator response^1^,^21^,^22^ and the effects of predator presence on group formation and group size have been demonstrated empirically in many studies^23^,^24^,^25^. The resulting group size of prey species has direct consequences for the behavioural activity of the group members. For example, in mixed-species flocks, the amount of scanning undertaken by individual birds –a measure of vigilance behaviour- decreases with increasing flock size^17^,^23^, allowing individuals to dedicate more time to active foraging^26^,^27^.

If individuals of a species differ in their vulnerability to predation or their foraging demands, starvation-predation risk trade-offs can result in differential foraging decisions in response to the presence of predators. For example, Cresswell^28^ observed than juvenile redshank (*Tringa totanus*) preferentially fed in more profitable but also more risky areas while adults avoided those risky areas. Similarly juvenile male dolphins (*Tursiops aduncus*) are more likely to accept high predation risk to achieve higher energy intake^29^. When disturbed by predators young red knots (*Calidris canutus*) were also more likely to resume foraging at the same place, while adults left the areas^30^. Sex is another source of inter-individual variation in foraging decisions under vaying predation risk which might even lead to sex-selective predation^31^. When resource availability is high, female skylarks (*Alauda arvensis*) are more likely to stay in a risky area while males will leave sooner^32^. In cercopithecoid primates the number of males in multi-male groups increases with increasing predation pressure, suggesting a trade-off between male reproductive success—which is higher for males that can monopolize females in single-male groups—and optimal group sizes to minimize the effects of predation^33^.

However, while predator presence has been shown to differentially affect foraging decisions and grouping behaviour of different phenotypes, the consequences of predation risk on the social relationships in foraging groups is still unknown. For example, social interactions in tit flocks are non-random and social relationships formed in these flocks are indicative for pair formation during the breeding season^34^,^35^,^36^,^37^. Changes in the social composition of these foraging flocks could, therefore, have consequences on the pair formation and, hence, mating success. Furthermore, individuals in foraging flocks might provide valuable information about population density and structure, habitat quality, or foraging opportunities, and this, too, will be affected by group composition.

In this study, we investigated how nonlethal predator effects influence the composition of winter flocks of foraging great tits (*Parus major*) and blue tits (*Cyanistes caerulaeus*). We determined whether simulated attacks of predators have a direct effect on flock stability and turn-over rates. We achieved this through exposing individually tagged tits at automated feeding stations to attacks by models of sparrowhawks—their natural predators—and comparing (a) individual responses and (b) changes in flock membership with control situations in the absence of predator threats. Specifically, we examined whether individuals were more likely to leave a feeding area after a simulated sparrowhawk attack, whether they took longer to return to the feeding area, and whether attacks increased the turn-over rates of birds present in the feeding area.

## Methods

### Study site and subjects

**Figure 1:**
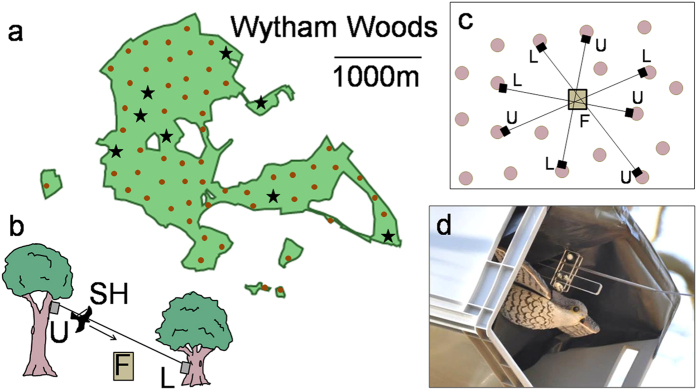
(**a**) Map of the study area Wytham woods, Oxfordshire (created in *Mathematica* 10.2 from Wolfram Research Inc., http://www.wolfram.com/). A grid of 65 seed feeders (brown dots) was deployed every weekend from September to February. Eight feeders (stars) were selected for simulated sparrowhawk attacks. (**b**) Sketch of a sparrowhawk unit, consisting of a sparrowhawk model (SH) and a steel cable running from an upper box (U) over the feeder (F) to a lower box (L). Sparrowhawks were released automatically upon detection of predefined PIT tagged individuals by the RFID antenna attached to the feeder. (**c**) At each experimental site we installed four sparrowhawk units next to a seed feeder, by fixing four upper boxes and four lower boxes to surrounding trees (grey disks), enabling up to four predator attacks per day. (**d**) Photo of a sparrowhawk model in an upper box. During the experiment the hawk model was hidden behind a curtain.

This field experiment was carried out in Wytham Woods (51°46′ N, 1°20′ W), a mixed deciduous woodland area of ca 385 ha, near Oxford, UK. Birds have been monitored in this area since the early 1960 s as part of a long-term population study^38^. The study species, great tits and blue tits, are small passerine birds which are territorial during the breeding season but form multi-species flocks – together with other (numerically much less abundant) tit species during the winter months^38^. Routine data collection during the breeding season (April–June) consists of nestbox checks to record breeding activity. Breeding adults and nestlings are ringed with uniquely numbered metal rings from the British Trust for Ornithology and standard morphometric information is recorded. In addition to the ringing effort during the breeding season, mist nets are used for catching and ringing non-breeding adults and immigrant birds throughout the winter. Since 2011 all breeding blue and great tits, and any birds caught in the non-breeding season were additionally fitted with plastic split rings containing passive integrated transponder tags (henceforth PIT-tags; from IB Technology, Aylesbury UK). It was estimated that over 90% of great tits in the area carry PIT tags^39^. For blue tits this proportion is probably slightly lower (see Supplementary Information). Each PIT tag contains a unique ten-character hexadecimal code that can be read by a radio frequency identification (RFID) antenna. A grid of 65 sunflower seed feeders that opened every weekend from September to February has been installed in the study area. Each seed feeder was fitted with an RFID antenna that recorded the time, place, and the identity of all visiting PIT-tagged individuals. Out of these 65 feeders, eight were selected for experimental treatment (experimental feeder, Fig. 1).

### Design of the model predator

The model predator consisted of a plastic bird model (length 350 mm, wingspan 560 mm) representing a flying male sparrowhawk; previous research has demonstrated that passerines perceive such models as potential predators, and respond with anti-predatory behaviour and increased vigilance that is not shown towards non-threatening objects of the same size^40^,^41^. Eurasian sparrowhawks, *Accipiter nisus*, are forest living raptors that hunt primarily on small passerine birds^42^. They are the principal predators of tits in the study area^43^. All models were hand coloured in order to closely match the natural colour patterns of sparrowhawks. Two rollers were fitted to the back of the sparrowhawk model, allowing it to run along a coated 2 mm steel cable. At each of eight selected experimental feeder sites we installed four predator units by fixing a plastic box (600 mm × 400 mm × 420 mm) to a tree at a height of 3.5 to 7 m and running a steel cable from the box over the seed feeder to a second box fixed to another tree at the other side of the feeder at a height of 1 to 2 m (Fig. 1). The boxes and cables were attached to the trees two weeks before the start of the experiment and left there until the end of the study. The distance between the upper box and the lower box varied between 8 m and 17 m, with an inclination between 8° and 26°. The open side of each box was concealed by a set of dark green curtains. During the experiment, the hawk model was placed in the upper box, hidden behind the curtain, where it was held in place by a small hook. A solenoid fitted within the box was positioned to lift the hook upon activation. Triggering the solenoid therefore allowed the hawk model to run down the steel cable (driven by gravity), making an ‘attack’ by swooping over the feeder and landing in the lower box, where it remained concealed behind a curtain for the rest of the day. Releases were triggered automatically via an electronic circuit board (Stickman Technology Inc., Southampton) that was connected to the RFID antenna at the feeder, thus allowing the sparrowhawk to be released upon the RFID antenna detecting a given PIT tag. The speed of the model hawks over the feeders varied between approximately 4 and 11 ms^−1^, which is slower than real sparrowhawks during attacks (up to 16 ms^−1^)^42^, though the strong response of the tits, along with previous research demonstrating the effectiveness of these models^40^, make us confident that the sparrowhawk models were perceived as a threat. Models were only maintained and set by the experimenters between 19:00 and 21:00 h (outside of bird feeding times) to avoid experimenters causing disturbance. Thus, on each feeder site, the maximum number of attacks per day was limited to four: one on each line.

### Experimental procedure

Experiments were conducted on consecutive weekends from 09/02/2013 to 04/03/2013 (season 1) and 15/11/2013 to 23/02/2014 (season 2) with the exception of 27–29/12/2013. The experimental protocol differed slightly between seasons. During season one, six experimental feeders were opened on Friday and all visits of PIT tagged birds were logged. Out of all birds visiting the feeder on that day we randomly selected five potential ‘target birds’ for the next day. Saturday was an experimental treatment day, whereby the feeder’s circuit board was programmed to release a model sparrowhawk (with a probability of 0.05) upon the RFID antenna detecting a target bird. Once the first hawk was released, further hawks were only released by visits of the same focal individual (i.e. it became the sole target bird) for that weekend. Once birds were chosen as target birds they were excluded from the list of potential target birds for all following weekends. The motivation for this selection procedure was to distribute releases of hawks more evenly over the day and to ensure that all birds had a similar chance of experiencing an attack. By conditioning the release on the presence of a bird at a feeder, we avoided ‘null releases’ at times when no birds were near the feeder as well as ultimately simulating more realistic attacks, as hawks whooshed over the feeders only when a bird was perched upon it. On Sunday the birds were subjected to the same experimental treatment with the same target bird as on Saturday. On Saturday and Sunday all 65 seed feeders in the study area were open and logged bird visits. On Monday, experimental feeders were opened again for logging of post-experimental activity but no sparrowhawks were released on that day. During the second season of the study, the procedure differed in that there was only one day of predator attacks per weekend, with pre-experimental logging at five experimental feeders on Friday, predator treatment on Saturday and post-experimental control logging on Sunday. To allow combined analysis of season 1 and 2, we excluded the second day of experimental treatment from season 1 (thus excluding 19 model hawk releases) from further analysis, therefore analysing one day of predator treatment over all weekends. Overall, sparrowhawk models were released at 8 different feeder sites on 22 different weekends, resulting in a total of 112 sparrowhawk attacks.

### Identifying Flocks

Feeding activity at feeders is highly variable over time and clearly deviates from expectations for random activity^35^. During winter months, tits form mixed species foraging flocks^44^,^45^ and burst of activity at a feeder signify the arrival of such a flock. We used a machine learning algorithm based on a Gaussian mixture model^35^,^46^ for identifying instances of flocking. This algorithm searches for temporal regions of increased activity at the feeder and divides the continuous time stream of recorded visits into discrete gathering events, reflecting those ‘bursts’ of activity. Gathering events lasted between 1 and 2373 s (median = 208 s, IQR: 109–295 s) and comprised between 1 and 28 birds (median = 4, IQR: 2–7). For each gathering event we assumed a ‘gambit of the group’ approach^47^ by considering that every bird that visited the feeder during this gathering event at least once was associated with every other bird detected during the same gathering event at the same feeder.

### Demography and sample

Blue tits and great tits that were fitted with PIT tags and were recorded by the antenna at the feeder during a gathering event were considered as being present during this gathering event. Further, birds might have been present without showing up in the data set if they (i) were members of non-focus species, (ii) did not carry PIT-tags, or (iii) were present in the area of the feeder but did not land on the antenna. Thus, we acknowledge that the detected birds represent only a sample of the total number of present birds, and the actual number of birds might have been higher than the reported numbers in all conditions. We assume that all birds present during a gathering event with a model sparrowhawk release are likely to notice this event. The 112 attacks were, thus, experienced by 281 different birds. Out of these birds 129 (46%) experienced only a single attack while the remaining birds were present repeatedly (median 2 times, IQR: 1–3), totalling up to 705 observed responses of birds to model predator attacks. In those cases where birds experienced more than one attack, the median time gap between attacks was 7 days. Great tits made up 60% of the birds at the feeders during attacks and blue tits 40% (only these two species were considered). As those tits forage and roam in mixed-species flocks^44^,^45^, where individuals of both species freely mix and interact with each other, we performed all tests on the combined data set of both species. Sexes were almost equally represented with 38% males, 34% females and 28% birds of undetermined sex (yearlings that were not subsequently seen breeding). Most of the birds (68%) were yearlings –i.e. birds that hatched in the previous breeding season, while the remaining 32% were adults.

### Measures of turn-over

In order to quantify the change in group composition between successive gathering events we calculated three measures. The index *change (I*_*c*_) relates the number of individuals that were present in both gathering events to the number of individuals that were present in either event as


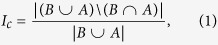


where *B* (as in “Before”) is the set of individuals present in the first gathering event and *A* (as in “After”) is the set of individuals present in the second gathering event. The term 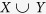 denotes the union of the two sets *X* and *Y*, the term 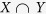 denotes the intersection of the sets *X* and *Y*, the term *X*\*Y* denotes the complement or difference set of all those elements in *X* that are not elements of *Y* and |*X*| is the cardinality of the set X, i.e. the number of elements in *X*. That is, the index *change* gives the proportion of individuals that were not present in both events. The index *blending (I*_*b*_) relates the relative number of individuals which were present in both events to the number of individuals present in either event and is given by


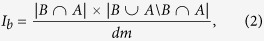


where *dm* = (|*B*∪*A*|/2)^2^ if |*B*∪*A*| is even and 
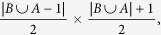
 otherwise. In other words, blending gives a value of zero if there is either no or complete overlap in the animals present before and after an attack and a value of one if those animals present both before and after the attack comprise 50% of all birds present. Finally, we calculate a *mixing* index (*I*_*m*_) as *I*_*m*_ = 1−*P*_*m*_ where *P*_*m*_ gives the probability of getting such an overlap, or one smaller than the observed one, if all individuals that were recorded during either of the gathering events were in the surrounding of the feeder for the whole period in question and visiting the feeder at random. *P*_*m*_ is calculated as the tail probability of getting the observed or a smaller intersection of two sets *A* and *B* of size *a* and *b*, respectively, by independently sampling first *a* and then *b* elements from a pool of *u* elements, which is given by


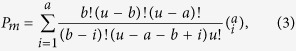


with *a* = |*A*|, *b* = |*B*|, and *u* = |*B*∪*A*|. For all three indices, a value of zero indicates perfect overlap, which is achieved when exactly the same set of individuals was observed in both gathering events. For the *change* indices a value of one indicates complete turnover with none of the individuals present in the first event being also present in the second event. The mixing index takes into account that an overlap of zero could also be due to chance if birds visit the feeder at random, though as the probability of a null overlap might be relatively small (depending on the total number of birds present), the *mixing* index will be close to one in such a case. For the blending index a value of one indicates maximal blending while zero indicates either perfect overlap or no overlap of individuals between the before and after events. Graphical explanations of the turn-over indices are given in Supplementary Figs S5 and S6 and a graphical illustration of how the three turnover indices compare to each other is provided in Supplementary Fig. S7.

### Statistical analysis

We used a matched control design, where each experimental treatment was matched with two control treatments at the same location and at a comparable time of the day (time-matched control): a pre-experimental control on the day before the attack and a post-experimental control on the day after the attack. If it was not possible to take the control from days directly before or after the attack (in a few cases there were no recordings on those days), we made controls at appropriate dates as close in time to the attack date as possible (see Supplementary Table 4a–c for details). With this matching procedure we control for seasonal differences (as every attack event is matched with a gathering event close in time), local differences in feeder popularity (as we match each attack with a gathering event at the same feeder) and differences in foraging activity along the course of the day (as we matched events at comparable daytimes).

For Individual-level analyses of the birds’ responses we employed general linear mixed model (GLMM) comparisons. For the latency to return after an attack (or matched control) we used a linear model with identity link, Gaussian error term, condition (attack or control), season, age class (yearling or adult), species (great tit or blue tit), time of the day, the number of previously experienced attacks and numbers of birds present during the attack as fixed factors and feeder location, weekend and bird identity as random factors. The full model was compared with a restricted model without the factor condition with a likelihood ratio test on the model deviances. For the binary outcome variables, i.e. whether a bird returned after an attack to the same feeder at that day (“return”) and whether a bird returned after an attack during the first post-attack gathering event (“immediate return”), we used generalized linear mixed models with logit link functions and binomial error terms and the same predictor variables as before. This analysis was carried out with the statistical software R^48^, using the functions lmer and glmer of the package lme4^49^. Parameters were estimated using the Maximum Likelihood method.

In addition to an analysis of individual responses we performed a group-level analysis where the main response variables of interest were measures of group turn-over before and after the simulated predator attack. We employed the same time-matched control design as for the individual-level analysis, yet as group composition in mixed species tit flocks changes quickly^50^,^51^, differences in the composition of groups between subsequent gathering events after hawk attacks and after the matched control events could be due to the longer temporal delay of the former. We therefore introduced a second set of matched controls. In this, we matched each attack event with another gathering event on a previous or successive day at the same feeder, where the time gap between the matched control event and its follow-up event was as close as possible to the time gap between the attack event and its follow-up event (gap-matched control). With this gap-matched control we created a set of control samples where the median gap times to the successive gathering events were 440 s (IQR: 257–771 s) for the pre-attack control and 437 s (IQR: 270–787 s) for the post-attack control, which was very close to the median gap-time between the attack and follow-up events of 451 s. Having controlled for temporal and local variation in bird activity by design, we used Wilcoxon tests for paired samples to compare responses in experimental and control treatments.

As this study was a field experiment with unrestricted access to the feeder, individual identity of the birds present was beyond our control. Overall, 46% of the birds recorded during the experiment experienced only a single attack event and the median number of times birds were present during attacks was two. For a field study with unrestricted access this is, in our opinion, a low level of repeated measures on the individual level and we can say that differences in experience with the model hawks were modest. More importantly, for the measures of group turn-over the level of analysis is in our case the foraging flock, as we measure the time gap between flocking events and the proportional change in flock composition. In total we had only a single instance (out of 112 attacks), where the same combination of individuals was present twice. We argue that this is a level of repeated measures that can safely be ignored for the kind of group-level analysis provided below. Throughout the text we give median values together with the 50% inter-quartile range (IQR). We performed multiple significance tests (Wilcoxon tests for paired samples), as we compared the response to an attack both with a pre- and a post-treatment control. As diverging results for pre- and post-treatment comparisons would have led to different conclusions, we recommend interpreting the reported p-values of those tests against a Bonferroni corrected significance level α′ = 0.016.

### Ethical statement

All work was subject to review and approved by the local ethical review committee at the Department of Zoology (University of Oxford) and also in accordance with the UK standard requirements and the ASAB guidelines for the use of animals in research. The work was conducted as part of a larger ongoing research project at Wytham woods, and all birds were caught, tagged and ringed by appropriate BTO licence holders.

## Results

### Behavioural responses

Birds reacted strongly to the attacks of the model sparrowhawks and usually left the feeder within seconds after the release of the sparrowhawk model (median = 3 s, inter-quartile range, IQR: 2–3.5 s). In order to classify the birds’ responses to attacks by a model predator, we defined three response types: a bird was said to ‘return’ within the given day if the first observation of this bird after an attack was made at the same feeder where it witnessed the attack; a bird was said to ‘move’ within the given day if the first observation of this bird after an attack was made at a different feeder; and a bird was said to ‘disappear’ if the bird was not observed after the attack at any feeder for the rest of the day. In 522 cases (74%) a bird returned after an attack, while in 77 cases (11%) a bird moved to another feeder and in 106 cases (15%) the bird disappeared for the rest of the day (Supplementary Table S1). All our observations are restricted to those birds which were fitted with pit tags (estimated as roughly 90% of the population based on capture-recapture data^39^) and which visit the feeders. The time gap between the end of the attack event and the subsequent gathering event was more than twice as long (median = 451 s, IQR: 257–877 s) than the time gap between time-matched control events and their successive gathering event (pre-attack: median = 131 s, IQR: 26–325 s, post-attack: median = 164 s, IQR: 54–356 s). These differences are both significant (Wilcoxon tests for paired samples, pre-attack: N = 112, T_s_ = 744, p < 0.001, post-attack: N = 112, T_s_ = 1267, p < 0.001).

### Individual-level effects

In order to investigate whether an ‘attack’ by a model sparrowhawk had an effect on the likelihood of a bird returning to the same feeder after the attack, we used a likelihood ratio test for comparing the deviances of a full GLMM and a restricted model not containing the experimental condition (attack versus control). Birds were less likely to return on the same day to the feeder where they witnessed an attack than were birds in both pre- or post-attack control trials (Δ_Dev_ = 84.0, df = 1, p < 0.001, and Δ_Dev_ = 75.6, df = 1, p < 0.001, Supplementary Table S3). Birds were also less likely to be detected in the first gathering event after an attack at the same feeder than were birds in both pre- or post-attack control trials (Δ_Dev_ = 93.6, df = 1, p < 0.001, and Δ_Dev_ = 91.3, df = 1, p < 0.001). Finally, those birds that did return to the feeder after an attack did so only after a longer time delay compared to the pre- and post- attack matched control conditions (Δ_Dev_ = 202.7, df = 1, p < 0.001, and Δ_Dev_ = 132.9, df = 1, p < 0.001, respectively).

### Flock composition

**Figure 2:**
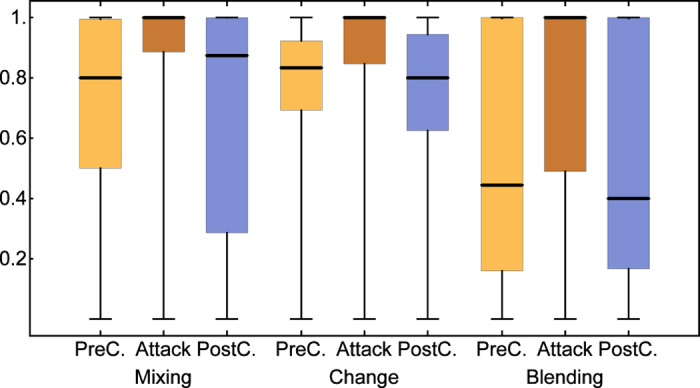
Time-matched comparisons for group turn-over. Three measures of turn-over in group composition in the experimental condition after a simulated attack (Attack), in a pre-treatment matched control condition (without a sparrowhawk model release) at the same feeder and time of day on a day before the attack (PreC), and in post-treatment control at the same feeder and time of day on a day after the attack (PostC). Indices of zero indicate no change in group composition and indices of one or close to one indicate complete turn-over with no overlap in group membership (see text for definitions for the measures). Bold bars give the median, boxes the inter-quartile range, and whiskers the range.

**Figure 3:**
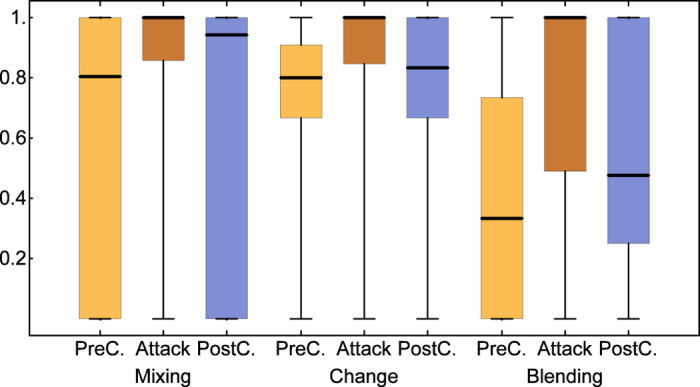
Gap-matched comparisons for group turn-over. Three measures for turn-over in group composition in the experimental condition after a simulated attack (Attack) and in two gap-matched control conditions without sparrowhawk release at the same feeder on a day before the attack treatment (PreC) and the day after the attack treatment (PostC) with a comparable time gap between control event and follow-up event to the time gap between attack event and the follow-up event in the *Attack* condition. Indices of zero indicate no change in group composition while indices of one or close to one indicate complete turn-over with no overlap in group membership, or maximal blending; bold bars give the median, boxes the inter-quartile range, and whiskers the range.

Comparing the number of birds in the first post-attack gathering event we found fewer birds at feeders after model hawks attacked (median = 2, IQR: 1–4) than in both the time-matched control condition (pre-attack: median = 4, IQR: 2–7, Wilcoxon test for paired samples: N = 112, T_s_ = 3695, p < 0.001, post-attack: median = 5, IQR: 2–8, N = 107, T_s_ = 3003, p < 0.001), and the gap-matched control (pre-attack: median = 3, IQR:1–7, N = 112, T_s_ = 2967, p = 0.003, post-attack: median = 3, IQR:1–6, N = 112, T_s_ = 2187, p = 0.013). Comparing birds present during the attack events and the post-attack gathering events, 26% of the birds present during the attack were not present afterwards. In order to investigate whether this corresponds with the expected value given continuous changes in group composition, we calculated three indices quantifying different aspects of turnover in flocks (See Methods and Supplementary Figs S5–S7). We found that turn-over after model hawk attacks was significantly higher than in the time-matched control condition, and this was true for both pre- and post-experimental controls and all measures for turn-over (Wilcoxon tests for paired samples, pre-attack: N = 102, change: T_s_ = 895, p < 0.001, blending: T_s_ = 3089, p < 0.001, mixing: T_s_ = 902, p < 0.001, post attack: N = 98, change: T_s_ = 632, p < 0.001, blending: T_s_ = 2592, p < 0.001, mixing: T_s_ = 703, p < 0.001, Fig. 2). Furthermore, rates of turn-over after attacks were also significantly higher than in the gap-matched control condition (pre-attack: N = 103, change:, T_s_ = 674, p < 0.001, blending: 103, T_s_ = 2961, p < 0.001, mixing: 103, T_s_ = 479, p < 0.001, post-attack: N = 104, change: T_s_ = 882, p = 0.002, blending: Ts = 2139, p = 0.001, mixing: 842, p = 0.003, Fig. 3), suggesting that the higher turn-over is not only due to the increased latency to return after a model hawk attack.

**Figure 4:**
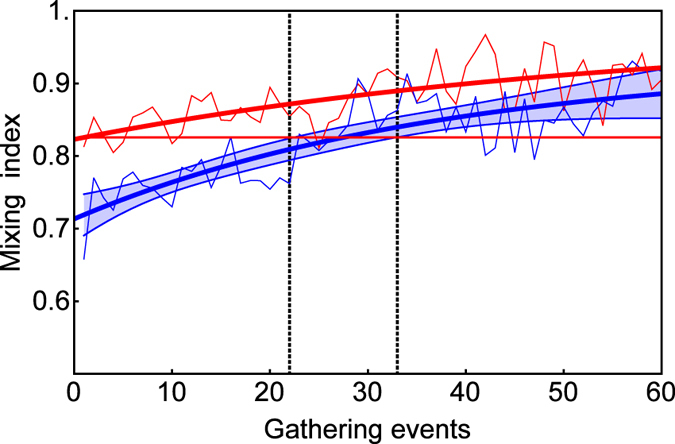
Mixing index for successive gathering events after an attack event in the experimental condition (red dot-dashed line) and after the matched control event in the post-experimental control condition (solid blue line). The bold line gives the fit of an exponential function to the mixing indices after all control events. The shaded area gives the 95% confidence interval for the estimated function for the control condition. Thin red and blue lines give the median values over all attack and control events. The horizontal line indicates the estimated mixing index for the first post-attack gathering event and the intersections between this line and the 95% CIs for the control condition were taken as the upper and lower estimates (dotted grey lines) for the time-effect that the attack had on the mixing index. On average, the distance between two consecutive gathering events equates to 6.5 minutes (392 s). Figures indicating the effect on the ‘blending’ and ‘change’ indices are provided in the online supplementary material.

As flock composition changes continuously, with birds leaving and other birds joining^50^, a gradual decrease in membership overlap with increasing time can be observed. Therefore, on finding that predator attacks induce instantaneous turn-over, we considered the amount of turnover, in terms of undisturbed foraging time, that a single predator attack generated. We calculated the three measures of turn-over (change, blending and mixing rates) for all subsequent flocking events at the respective feeder for the rest of the day. By fitting exponential functions to the observed indices, we were able to estimate the expected increase in the turn-over of group composition (Fig. 4). Based on this, we estimate that each single model predator attack generates the same change as we would otherwise expect, under normal conditions, after between 18.5 and 25 gathering events; the same blending expected after between 22.3 and 34.6 gathering events, and the same mixing as expect after between 22 and 33 gathering events. Given the distribution of gathering events in time (linear model fit to the median latencies for the first 35 post-event gathering events: *R*^2^ = 0.998, Supplementary Figure S9), this equates to the instantaneous change in group composition after a predator attack corresponding to the change expected after 2.0–2.6 hrs, the blending after 2.5–3.8 hrs and mixing index after 2.3–3.3 hrs, under standard, non-manipulated, conditions.

## Discussion

Using a large-scale field experiment, we demonstrate the nonlethal effects of simulated predator attacks on the behaviour of wild birds. As an immediate response to the sudden appearance of a model predator, the birds left the site. Birds were less likely to return to the site after an attack than in matched control situations and if they returned, they did so only after a considerably longer delay. As not all birds returned and new birds joined the flock of birds remaining at the feeder, this lead to an increased turn-over in group composition at the feeder site. The effect of an unsuccessful predator attack on the turn-over in group composition was rapid and extensive, and—depending on the chosen measure—equated to the turnover expected for 2.0–3.8 hrs of undisturbed foraging. Given a day length of eight to nine hours during winter, this corresponds to 22–47% of the time potentially available for foraging.

These estimates are associated with some uncertainty but, regardless of the measure of turn-over considered, the appearance of the model predator had a considerable effect on the turn-over within these flocks. As a caveat we want to mention the possibility that some of the undetected birds might have stayed in the vicinity of a feeder after an attack. A discussion of this scenario is given in the online supplementary material. An increased turn-over in group composition can affect the effective size of the social interaction network^52^ and, on the individual level, the number of potential interaction partners. It has been shown previously that changes in foraging associations in great tits carry over into other social contexts^53^,^54^. Increasing the number of interaction partners, and the rate at which these change, has the potential to influence many processes within populations with both negative and positive effects for individual fitness. For example, interacting with more conspecifics before the breeding season implies, on the one hand, meeting more potential mating partners and, on the other hand, meeting more competitors of the same sex. Both effects can have direct implications for an individual’s social behaviour and its future mating success. Further, the distribution of social interactions is often implicated–at least in theory–in emergent processes such as information or disease transmission, and recent work on tits has demonstrated effective social learning predicted by social structure^55^. Clearly, predator-driven changes in the rate of social association and turnover could have implications for such processes, even if this remains to be demonstrated empirically.

Predation is a strong mortality factor in mixed species tit flocks^56^ and sparrowhawks are amongst the main predators of tits in the study area^43^. Morse^57^ estimated that each individual tit may witness one sparrowhawk attack per day, with an estimated success rate of 12%^42^. Birds can reduce the risk of falling prey to hawks by increasing vigilance, staying closer to cover, moving less or hiding, though all these measures for evading predation also reduce foraging efficiency. That is, the birds have to trade off safety against foraging, and the balance between risks of predation and starvation is under constant selection pressure^3^,^40^,^58^,^59^,^60^,^61^. Whether a trade-off in tit flocks exists between safety and a favourable flock composition with respect to specific individuals remains unknown. Although we could not address this question with our study, such trade-offs between safety and social requirements could be elucidated by systematically altering the frequency of predator attacks, or by longitudinal studies if responses to predators change as the breeding season approaches. Finally, pair formation, which intensifies in spring, might require that pair members condition their decision (e.g. whether to stay or to go) on the response of their partner. As a recent study has shown that great tits opt to forage at sub-optimal patches in order to stay together with their preferred social partner^53^, it seems plausible that decisions about leaving an area due to increased predator presence might be influenced by the partner’s response as well. We observed some instances where mated pairs of birds were present during attacks (summarized in Supplementary Table 1), but the small number of these occasions prohibited any analysis in this direction.

In order to describe the predation risk effects on the behaviour and habitat use of prey species, Brown and colleagues^62^ coined the term “ecology of fear”. Here, we suggest extending this to a “socio-ecology of fear” when considering the effects of predation risk on social behaviour and group composition. Several empirical studies have shown that predation risk can influence individuals’ decisions to join groups^24^,^25^. Recently, Croft and colleagues^63^ found that under high predation pressure males guppies (*Poecilia reticulata*), which are more vulnerable to predation than females, form separate shoals in shallower and safer habitats, demonstrating that predation pressure can even lead to social segregation of the sexes. Predation risk effects on social behaviour are, however, not confined to foraging aggregations but have been debated for breeding colonies^64^, and empirical studies delivered evidence for such effects^22^,^65^,^66^.

Predators have direct impact on the population dynamics and individual behaviour of their prey species. The very evident and drastic impact of mortality caused by direct predation has led to most research primarily focusing on the consequences of elimination of individuals from the population. More subtle effects of predator presence have been studied predominantly in the context of foraging^61^,^67^. Here, we argue that nonlethal predator effects on social behaviour and social structure deserve more attention, as it is likely that they, in turn, have a strong influence on population level processes, as well as individual fitness by exerting sizeable influence on the social system and social interaction patterns of the prey species.

## Additional Information

**How to cite this article**: Voelkl, B. *et al.* Nonlethal predator effects on the turn-over of wild bird flocks. *Sci. Rep.* **6**, 33476; doi: 10.1038/srep33476 (2016).

## Supplementary Material

Supplementary Information
