# Supplementary material for: Nonlethal predator effects on the turn-over of wild bird flocks
Source: Sci Rep. 2016 Sep 16;6:33476. doi: 10.1038/srep33476 (PMC5025840; doi:10.1038/srep33476)
Supplement: Supplementary Information [file srep33476-s1.pdf]

**Nonlethal predator effects on the turn-over of wild bird flocks.**

Bernhard Voelkl, Josh A. Firth & Ben C. Sheldon

**Supplementary Information**

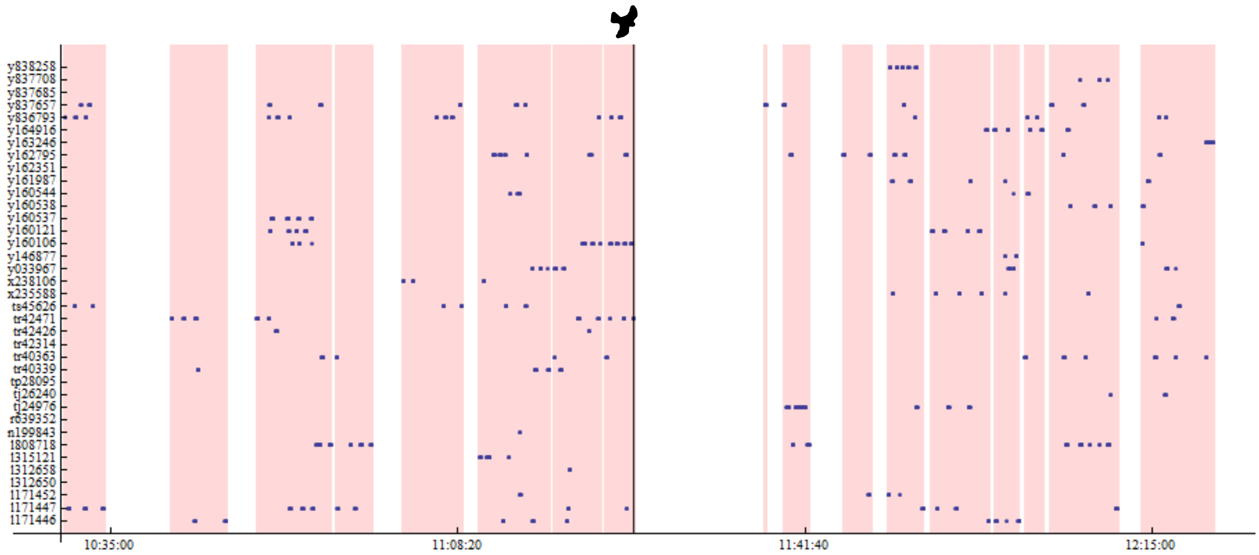


**Figure S1:** Visual representation of bird visits at a feeding station. Individual birds, indicated by their unique ring number, are aligned on the y-axis and time is plotted on the x-axis. This example shows data from a period of two hours at one feeder (4b) on March 3rd 2013. Single recordings of the birds' PIT tags by the RFID antenna attached to the feeder are indicated by blue dots. Using a machine learning algorithm based on a Gaussian mixture model we split the continuous data stream into discrete gathering events (indicated by pink shaded areas). White areas between gathering events were times where no bird was detected at the feeder. The release of a sparrowhawk model at 11:25:13 is indicated by a vertical black line.

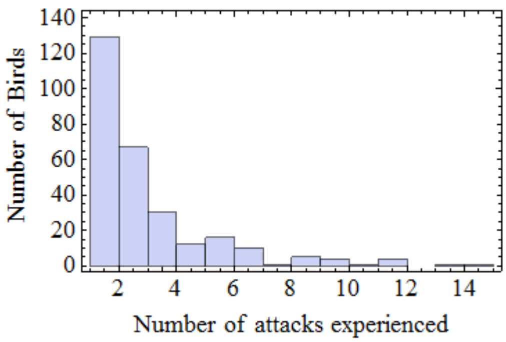


**Figure S2:** Histogram giving the distribution of the number of attacks experienced by each bird. 129 birds (46%) were present at only one gathering event when a hawk model was released. The median number of simulated attacks experienced by a single bird was 2, with an interquartile range of 1-3.

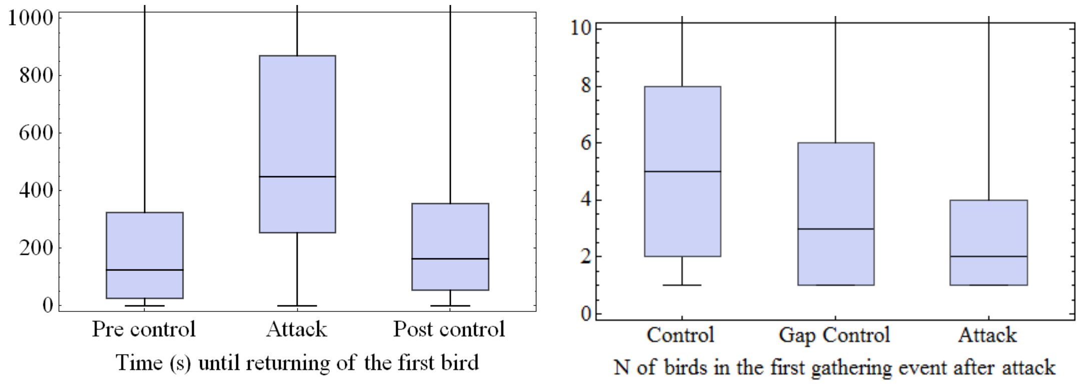


**Figure S3:** (a) Time gap between the gathering event when the attack happened (or the 'imagined attack' in the pre- and post-treatment control conditions) and the next gathering event afterwards at the same feeder. This is equivalent to the time between the last bird leaving the feeder following an attack and the first bird returning to the feeder thereafter. (b) Number of birds present in the first gathering event after an attack (or 'imagined attack').

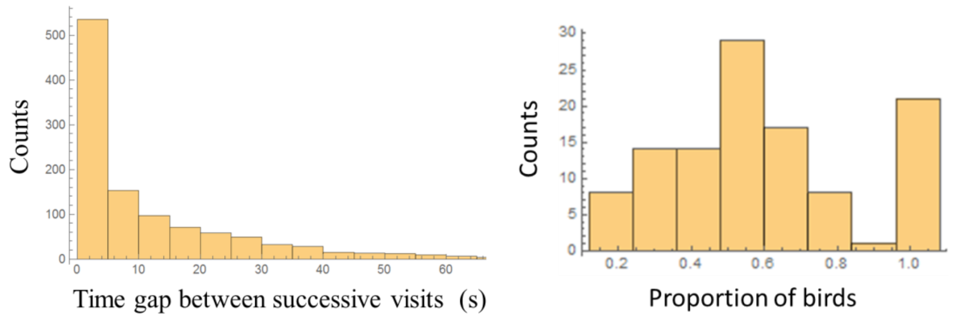


**Figure S4:** (a) Time gap between successive visits of the feeder by different birds during a gathering event. The median time between one bird leaving and another bird arriving at the antenna was 5s (IQR: 1, 17s). (b) Proportion of birds detected during the last minute during the attack gathering events.

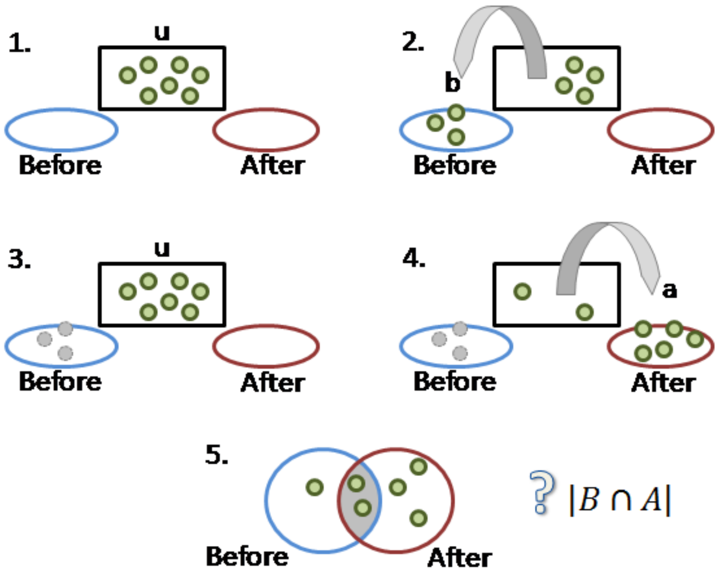


**Figure S5:** Visualization of the assumptions for the *mixing* index $I_m$. We make the null assumption that all birds in a set of individuals that were observed in either of two consecutive gathering events (termed "*Before*" and "*After*") are, in fact, in the vicinity of the feeder for the whole duration and that any differences in group composition of the two events is due to random sampling from the birds present, alone. This can be visualized as an urn problem (1), where we independently draw (without replacement) first *b* individuals from the set of *u* individuals (2), and thereafter from the full set of *u* individuals (3), again without replacement, *a* individuals (4). We want to know, how likely it is that we get, in such a case, an overlap $(Before \cap After)$ as large as the observed one (5). In other words, we want to know $E(|B \cap A|)|\{u, a, b\}$.

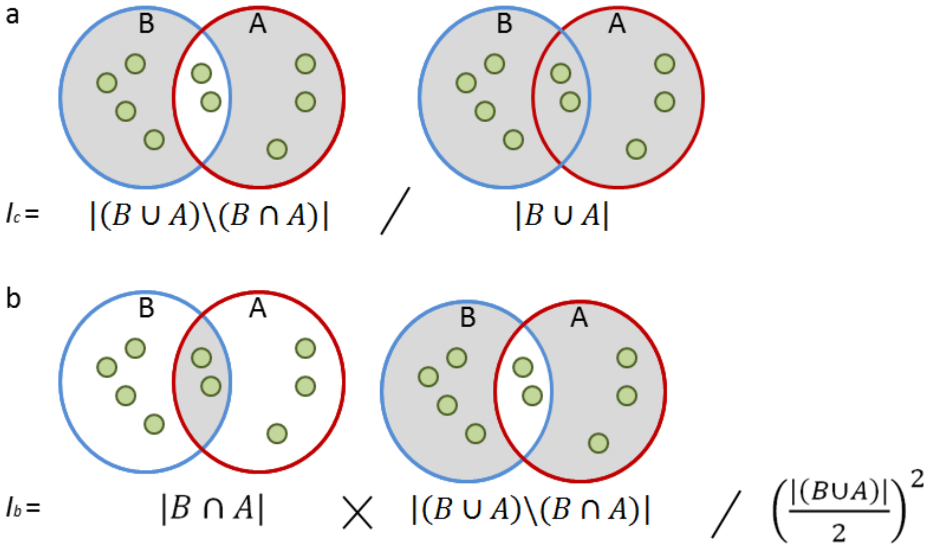


**Figure S6:** Venn diagram representation of (a) the *change* index $I_c$ and (b) the *renewal* index $I_r$. In the given example the *change* index $I_c$=7/9 and the *renewal* index $I_b$=7/10.

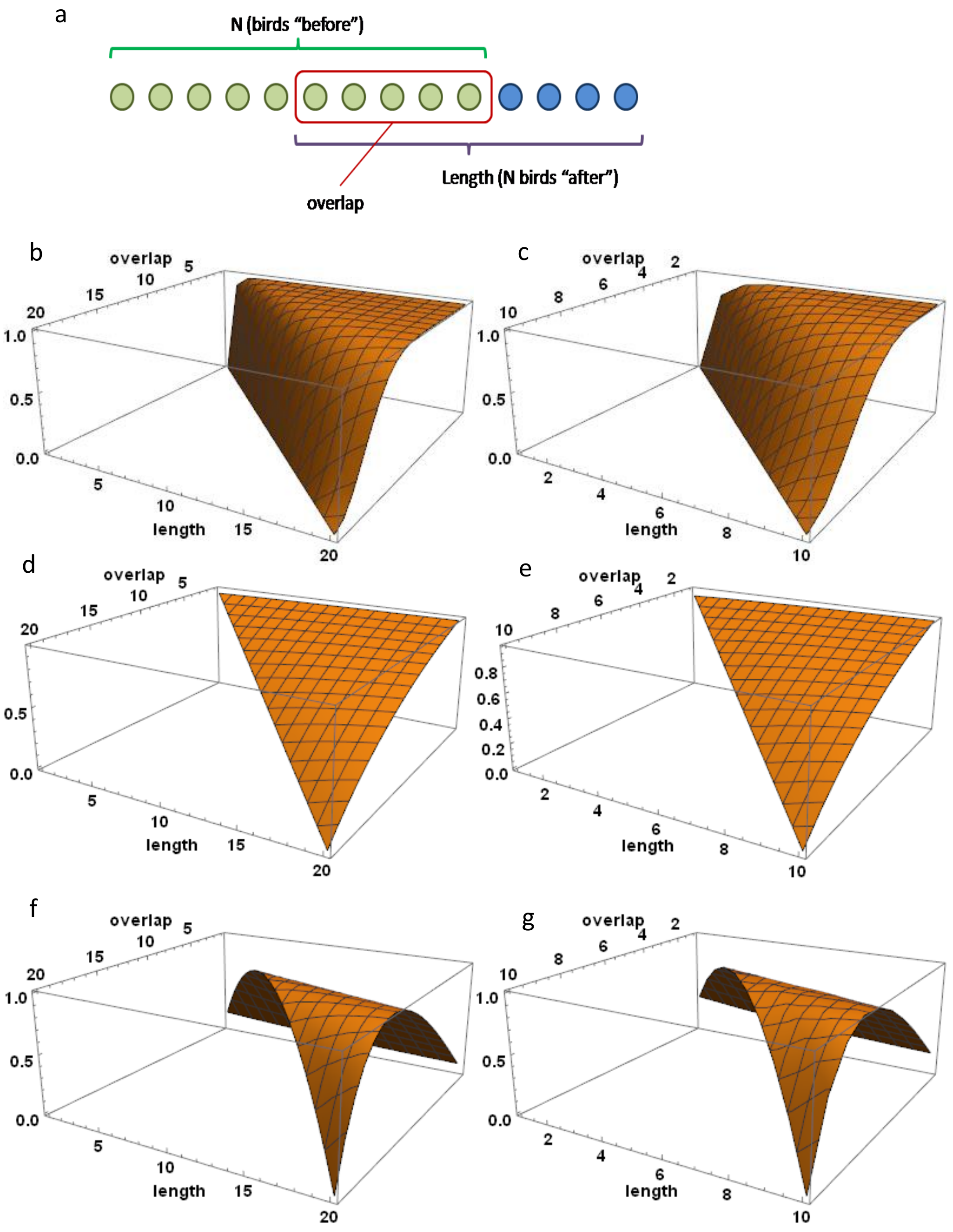


**Figure S7:** Comparison of the three indices for turn-over, $I_m$ (*mixing*), $I_c$ (*change*) and $I_b$ (*blending*). In order to visualize the behaviour of the three indices we plotted the indices for fixed group sizes of birds present during the first event ("before") of *N*=10 (b, d, f) and *N*=20 (c,e, g), with the group size of the birds present in the second event ("length") plotted on the x axis and the number of birds present in both events ("overlap") plotted on the y axis. (b) mixing index, $I_m$ , N=10, (c) mixing index, $I_m$ , N=20, (d) change index, $I_c$ , N=10, (e) change index, $I_c$ , N=20, (f) blending index, $I_b$ , N=10, (g) blending index, $I_b$ , N=20.

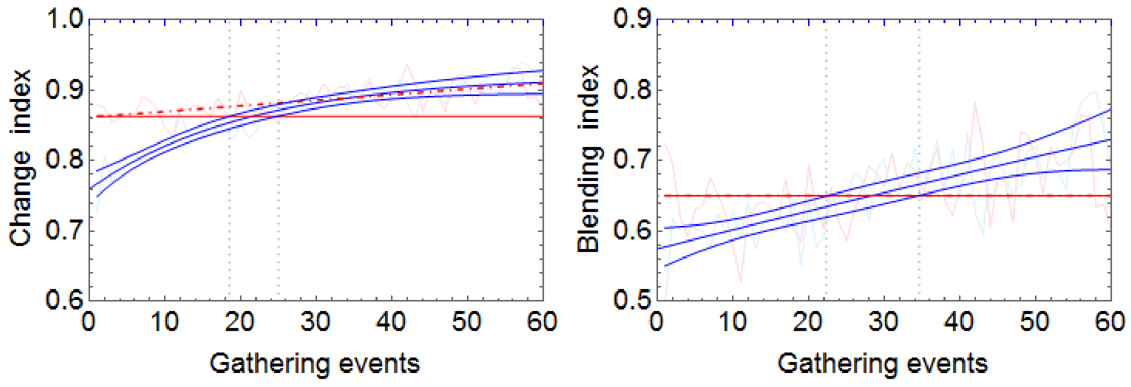


**Figure S8:** Change index $I_c$ (a) and blending index $I_b$ (b) for successive gathering events after an attack event in the experimental condition (red dot-dashed line) and after the matched control event in the post-treatment control condition (solid blue line). Bold lines give the fit of an exponential function to the indices after all experimental and control events respectively. The thin lines gives the 95% confidence interval for the estimated function for the control condition. Light red and blue lines give the median values over all attack and control events. The horizontal line indicates the estimated index for the first post-attack gathering event and the intersections between this line and the 95% CIs for the control condition were taken as the upper and lower estimates (dotted grey lines) for the time-effect that the attack had on the turn-over index.

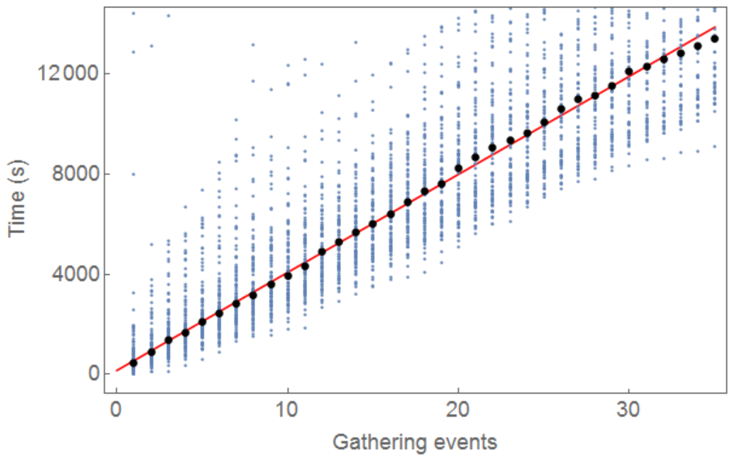


**Figure S9:** Waiting time between a matched control event and the subsequent 35 gathering events at the same feeder on the same day. Blue points give single observations, bold, black points median values. The red line is the regression line of a linear regression on the medians values ($R^2$=0.998, slope: 392s), indicating a linear relationship between event number and median waiting time. The waiting time for the 9$^{th}$ subsequent gathering event equates, therefore, to approximately 1 hour (3528s).

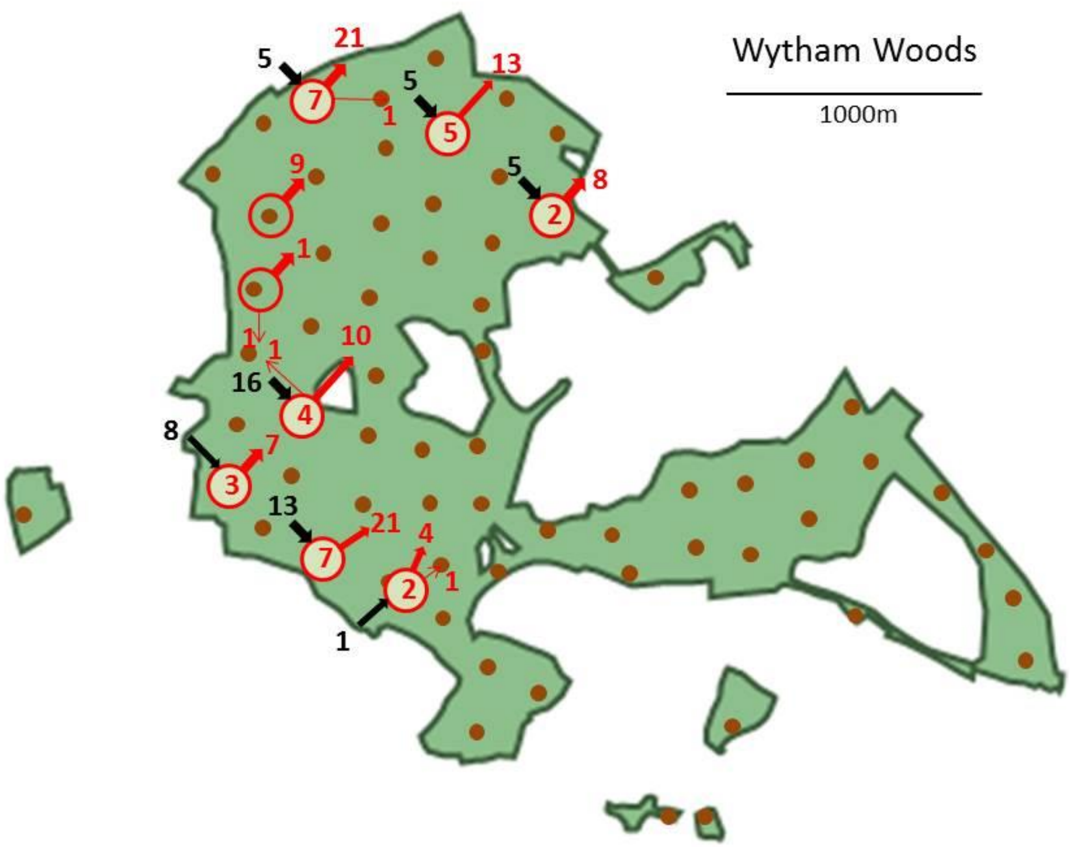


**Figure S10:** Results of a small-scale field experiment with two tame Harris's hawks (*Parabuteo unicinctus*) conducted on 27/01/2014. Two falconers (John Dowding and Jim Hill) from the International Centre of Birds of Prey, Gloucesteshire, UK, visited a set of selected feeders with two tame Harris's hawks. At each feeder, one falconer approached the feeder, stopping at a distance between 20 and 25m. The falconer then let the hawk fly to an assistant positioned approximately 40 away on the opposite side of the feeder. We recorded all visits of great tits and compared the identity of birds present during 15 minutes before the arrival of the Harris's hawk with the first 15 minutes after the first great tit returned to the feeder after the visit of the Harris's hawk. Red circles indicate feeders visited with the Harris's hawks. Red figures in the circles give the numbers of birds present both before and after the hawk visit, bold red arrows pointing away indicate numbers of birds that were present before the visit but not afterwards. Thin red arrows indicate birds moving to a different feeder after the visit. Green numbers with arrows pointing towards the circle indicate numbers of new arrivals, i.e. birds that were not present before the visit of the hawk, but joined the flock at the feeder afterwards. (Map created in Mathematica 10.2 from Wolfram Research Inc., http://www.wolfram.com/.)

**Supplementary Results**

Using automated feeding stations allows recording all birds fitted with pit tags and visiting the feeders, however we are blind to birds that are not fitted with pit tags (which are estimated to be less than 10 percent based on recapture rates during mist netting in the vicinity of feeders, see ref. 39) and we are also blind to birds that join flocks at feeders but do not take seeds from the feeder (as the antenna at the feeder reads only pit tags of birds landing on the perch in front of the feeder opening). In order to discuss how likely it is that birds are present in the area around the feeders but are not recorded we did the following: (1) We evaluated the proportion of birds found during the breeding season and fitted with pit tags in preceding years which were never recorded at feeders. Some of those birds might have left the area, as it is a frequently observed phenomenon that great tits and blue tits move closer to rural settlements during the winter, and such observations have also been made in the surrounding of Wytham woods. Yet, a part of those birds missing in the wither logging record might be still in the area but do not use the feeders. While we cannot distinguish between these two scenarios, the number of birds missing in the winter logging record can, at least, give an upper bound (though, quite likely an over-estimation) of the proportion of birds that are present but unrecognized. Out of the 999 pit-tagged birds (blue tits and great tits) recaptured during the breeding season 2014, 194 birds (19.4%) have not been recorded at feeders in the winter before. (2) We asked whether there are birds which are prevented from visiting the feeders by the presence of other birds. The presence of such 'subordinate' birds might inflate our estimates of new arrivals after attack events. (The 'new arrivals' might be just birds who were already in the area of the feeder but were prevented from visiting the feeders by the presence of other birds.) We therefore asked (a) whether there are birds who visit feeders only on their own, (b) whether there are birds which predominantly visit feeders in the second half of a gathering event, (c) whether those birds that 'moved in' after an attack were at other occasions seen together with those birds leaving the feeder. Out of the 3139 birds observed feeding at loggers only 4 birds were never seen to feed together (in the same gathering event) with other birds. Almost all birds were equally often seen in the first and the second half of a gathering event (average ratio: 0.499 ± 0.028 SD). For building this ratio we excluded those birds which were recorded in less than 10 distinct gathering events (as the ratio would be inconclusive for smaller counts), reducing our sample to 2794 birds. Only 15 out of those 2794 birds clearly deviated from a balanced ratio: 8 birds being seen more often in the first half of gathering events and 7 birds being seen predominantly in the second half of gathering events (based on individual $\chi^2$ tests with $\alpha$=0.05, though no significant deviations would be found when correcting for multiple testing). Out of the 1586 pairings of birds leaving a feeder after an attack and birds newly arriving at this feeder in the next gathering event, there were only 32 cases (2%) where those two birds were never observed together in a gathering event. This gives no indication that birds newly arriving after an attack are avoiding those birds which were leaving the feeder after an attack. Summarizing we can, therefore, say that the overall size of flocks (considering blue tits and great tits) might be up to a maximum of 30% larger than reported. However, this holds true for both pre- and post-attack gathering events and as such this does not affect the reported effects. We could not find indications that birds were excluded from visiting feeders at any phase of a gathering event or that newly arriving birds were avoiding those birds leaving a feeder.

**Supplementary Table 1: Individual responses to attacks**

| | Return | | Move | | Disappear | |
|---|---|---|---|---|---|---|
| | N | (%) | N | (%) | N | (%) |
| Great tits | 294 | (70) | 57 | (13) | 71 | (17) |
| Blue tits | 228 | (80) | 20 | (07) | 35 | (12) |
| | | | | | | |
| Males | 200 | (76) | 27 | (10) | 38 | (14) |
| Females | 170 | (71) | 21 | (09) | 48 | (20) |
| *unknown* | 152 | (76) | 29 | (14) | 20 | (10) |
| | | | | | | |
| Adults | 176 | (78) | 12 | (05) | 37 | (17) |
| Juveniles | 346 | (72) | 65 | (14) | 69 | (14) |
| | | | | | | |
| Breeding birds with partner present | 22 | (61) | 6 | (17) | 8 | (22) |
| Breeding birds with partner absent | 174 | (77) | 28 | (12) | 25 | (11) |
| Non-breeding birds | 326 | (74) | 43 | (10) | 73 | (16) |
| | | | | | | |
| Total | 522 | (74) | 77 | (11) | 106 | (15) |

Out of the 77 times that birds moved to another feeder it happened 10 times that a pair of birds moved to another feeder, one time three birds moved together and two times four birds moved together to another feeder. In two out of the 10 cases that two birds were moving to another feeder, the respective birds were breeding partners in the following breeding season. Breeding status refers to the breeding season after the experiment and ‘partner present’ or ‘partner absent’ to the presence or absence of the breeding partner during the gathering event in which the attack happened.

**Supplementary Table 2a:** Individual responses in the post-attack matched control condition.

| | Return | | Move | | Disappear | |
|---|---|---|---|---|---|---|
| | N | (%) | N | (%) | N | (%) |
| Great tits | 228 | (94) | 2 | (1) | 13 | (5) |
| Blue tits | 238 | (90) | 9 | (3) | 18 | (7) |
| | | | | | | |
| Males | 161 | (89) | 6 | (3) | 14 | (8) |
| Females | 164 | (96) | 2 | (1) | 5 | (3) |
| *unknown* | 141 | (90) | 3 | (2) | 12 | (8) |
| | | | | | | |
| Adults | 159 | (93) | 4 | (2) | 8 | (5) |
| Juveniles | 307 | (91) | 7 | (2) | 23 | (7) |
| | | | | | | |
| Breeding birds with partner present | 23 | (96) | 0 | (0) | 1 | (4) |
| Breeding birds with partner absent | 181 | (90) | 3 | (2) | 17 | (8) |
| Non-breeding birds | 262 | (92) | 8 | (3) | 13 | (5) |
| | | | | | | |
| Total | 466 | (92) | 11 | (2) | 31 | (6) |

**Supplementary Table 2b:** Individual responses in the pre-attack matched control condition.

| | Return | | Move or Disappear | |
|---|---|---|---|---|
| | N | (%) | N | (%) |
| Great tits | 283 | (92) | 23 | (8) |
| Blue tits | 239 | (95) | 12 | (5) |
| | | | | |
| Males | 203 | (91) | 20 | (9) |
| Females | 176 | (96) | 8 | (4) |
| *unknown* | 143 | (95) | 7 | (5) |
| | | | | |
| Adults | 135 | (94) | 8 | (6) |
| Juveniles | 387 | (93) | 27 | (7) |
| | | | | |
| Breeding birds with partner present | 33 | (97) | 1 | (3) |
| Breeding birds with partner absent | 207 | (95) | 11 | (5) |
| Non-breeding birds | 282 | (92) | 23 | (8) |
| | | | | |
| Total | 522 | (94) | 35 | (6) |

Note that for the pre-attack matched control (on Fridays) it was not possible to distinguish between moving and disappearing, as no data logging was taking place at feeding stations other than the targeted feeders.

**Supplementary Table 3a:** Return on the same day: Attack vs. pre-attack matched control.

| **Model** | ***df*** | ***log* link** | **AIC** | **BIC** | **Deviance** | **Δ*Dev*** | **LRT *df*** | **LRT *p* ($X^2$)** |
|---|---|---|---|---|---|---|---|---|
| Full | 11 | -439.4 | 900.8 | 957.2 | 878.8 | 84.0 | 1 | < 2e-16 |
| Restricted | 10 | -481.4 | 982.8 | 1034.1 | 962.8 | | | |

| **Random effects** | **Obs** | **Variance** | **Std** | |
|---|---|---|---|---|
| Bird ID | 356 | 2.235e-06 | 0.001495 | |
| Weekend | 21 | 0.3404 | 0.583470 | |
| Logger | 8 | 0.1991 | 0.446203 | |

| **Fixed effects** | **Estimate** | **SE** | ***z*** | ***p* (*X*>*z*)** |
|---|---|---|---|---|
| Intercept | 1.87408 | 0.41932 | 4.469 | 7.85e-06 |
| Condition | 1.83773 | 0.22432 | 8.193 | 2.56e-16 |
| Age | 0.37096 | 0.25351 | 1.463 | 0.14338 |
| Species | -0.53708 | 0.19764 | -2.717 | 0.00658 |
| Season | -0.58168 | 0.44536 | -1.306 | 0.19152 |
| Time | -1.06217 | 0.11895 | -8.930 | < 2e-16 |
| N birds | 0.24322 | 0.11357 | 2.142 | 0.03223 |
| Attack | 0.03641 | 0.03486 | 1.044 | 0.29633 |

Generalized linear mixed effect model with *Logit* link and binomial error term. Number of observations: 1251. Estimates based on maximum likelihood approximation. Δ*Dev:* deviance difference between the full model and the restricted model without condition , LRT: likelihood ratio test, df: degrees of freedom, Std: standard deviation. BirdID: individual bird identity, Weekend: experimental weekend (replicate), Logger: location, Condition: attack vs. control, Age: adult vs. young, Species: great tit vs blue tit, Season: experimental season 2012/13 vs. 2013/14, Time: time of the day, attack: number of attacks previously experienced by the bird.

**Supplementary Table 3b:** Return on the same day: Attack vs. post-attack matched control.

| **Model** | ***df*** | ***log* link** | **AIC** | **BIC** | **Deviance** | **Δ*Dev*** | **LRT *df*** | **LRT *p* ($X^2$)** |
|---|---|---|---|---|---|---|---|---|
| Full | 11 | -448.1 | 918.2 | 974.3 | 896.2 | 75.6 | 1 | < 2e-16 |
| Restricted | 10 | -485.9 | 991.8 | 1042.8 | 971.8 | | | |

| **Random effects** | **Obs** | **Variance** | **Std** | |
|---|---|---|---|---|
| Bird ID | 336 | 4.934e-10 | 2.221e-05 | |
| Weekend | 21 | 0.2749 | 0.5243 | |
| Logger | 8 | 0.4981 | 0.7057 | |

| **Fixed effects** | **Estimate** | **SE** | ***z*** | ***p* (*X*>*z*)** |
|---|---|---|---|---|
| Intercept | 1.66938 | 0.43529 | 3.835 | 0.00013 |
| Condition | 1.70485 | 0.22360 | 7.982 | 1.44e-15 |
| Age | 0.04084 | 0.23666 | 0.173 | 0.86299 |
| Species | -0.12556 | 0.19226 | -0.653 | 0.51371 |
| Season | -0.65333 | 0.41463 | -1.576 | 0.11510 |
| Time | -0.85750 | 0.10599 | -8.091 | 5.93e-16 |
| N birds | 0.64501 | 0.12097 | 5.332 | 9.71e-08 |
| Attack | 0.04743 | 0.03364 | 1.410 | 0.15862 |

Generalized linear mixed effect model with *Logit* link and binomial error term. Number of observations: 1213. Estimates based on maximum likelihood approximation. Δ*Dev:* deviance difference between the full model and the restricted model without condition , LRT: likelihood ratio test, df: degrees of freedom, Std: standard deviation. BirdID: individual bird identity, Weekend: experimental weekend (replicate), Logger: location, Condition: attack vs. control, Age: adult vs. young, Species: great tit vs blue tit, Season: experimental season 2012/13 vs. 2013/14, Time: time of the day, attack: number of attacks previously experienced by the bird.

**Supplementary Table 3c:** Return during the next gathering event: Attack vs. pre-attack matched control.

| Model | *df* | *log* link | AIC | BIC | Deviance | Δ*Dev* | LRT *df* | LRT *p* ($X^2$) |
|---|---|---|---|---|---|---|---|---|
| Full | 11 | -587.8 | 1197.6 | 1254.0 | 1175.6 | 93.6 | 1 | < 2e-16 |
| Restricted | 10 | -634.6 | 1289.2 | 1340.5 | 1269.2 | | | |

| Random effects | Obs | Variance | Std |
|---|---|---|---|
| Bird ID | 356 | 0.19411 | 0.4406 |
| Weekend | 21 | 0.15660 | 0.3957 |
| Logger | 8 | 0.06978 | 0.2642 |

| Fixed effects | Estimate | SE | *z* | *p* (*X*>*z*) |
|---|---|---|---|---|
| Intercept | -2.36920 | 0.32653 | -7.256 | 4.00e-13 |
| Condition | 1.49988 | 0.16376 | 9.159 | < 2e-16 |
| Age | 0.42936 | 0.21834 | 1.967 | 0.0492 |
| Species | 0.09998 | 0.17240 | 0.580 | 0.5619 |
| Season | -0.06792 | 0.33788 | -0.201 | 0.8407 |
| Time | 0.02541 | 0.08510 | 0.299 | 0.7652 |
| N birds | -0.36304 | 0.09196 | -3.948 | 7.89e-05 |
| Attack | 0.00931 | 0.03020 | 0.309 | 0.7577 |

Generalized linear mixed effect model with *Logit* link and binomial error term. Number of observations: 1251. Estimates based on maximum likelihood approximation. Δ*Dev:* deviance difference between the full model and the restricted model without condition *,* LRT: likelihood ratio test, df: degrees of freedom, Std: standard deviation. BirdID: individual bird identity, Weekend: experimental weekend (replicate), Logger: location, Condition: attack vs. control, Age: adult vs. young, Species: great tit vs blue tit, Season: experimental season 2012/13 vs. 2013/14, Time: time of the day, attack: number of attacks previously experienced by the bird.

**Supplementary Table 3d:** Return during next gathering event: Attack vs. post-attack matched control.

| Model | *df* | *log* link | AIC | BIC | Deviance | Δ*Dev* | LRT *df* | LRT *p* ($X^2$) |
|---|---|---|---|---|---|---|---|---|
| Full | 11 | -583.8 | 1189.8 | 1245.9 | 1167.8 | 91.3 | 1 | < 2e-16 |
| Restricted | 10 | -629.6 | 1279.1 | 1330.1 | 1259.1 | | | |

| Random effects | Obs | Variance | Std |
|---|---|---|---|
| Bird ID | 336 | 0.008488 | 0.09213 |
| Weekend | 21 | 0.027851 | 0.16689 |
| Logger | 8 | 0.191447 | 0.43755 |

| Fixed effects | Estimate | SE | *z* | *p* (*X*>*z*) |
|---|---|---|---|---|
| Intercept | -1.650848 | 0.278391 | -5.930 | 3.03e-09 |
| Condition | 1.472353 | 0.163175 | 9.023 | < 2e-16 |
| Age | -0.080483 | 0.196833 | -0.409 | 0.6826 |
| Species | 0.103080 | 0.161673 | 0.638 | 0.5237 |
| Season | -0.515749 | 0.258189 | -1.998 | 0.0458 |
| Time | -0.004939 | 0.078855 | -0.063 | 0.9501 |
| N birds | -0.149854 | 0.097269 | -1.541 | 0.1234 |
| Attack | -0.024057 | 0.027490 | -0.875 | 0.3815 |

Generalized linear mixed effect model with *Logit* link and binomial error term. Number of observations: 1213. Estimates based on maximum likelihood approximation. Δ*Dev:* deviance difference between the full model and the restricted model without condition*,* LRT: likelihood ratio test, df: degrees of freedom, Std: standard deviation. BirdID: individual bird identity, Weekend: experimental weekend (replicate), Logger: location, Condition: attack vs. control, Age: adult vs. young, Species: great tit vs blue tit, Season: experimental season 2012/13 vs. 2013/14, Time: time of the day, attack: number of attacks previously experienced by the bird.

**Supplementary Table 3e:** Time to return: Attack vs. pre-attack matched control.

| Model | *df* | *log* link | AIC | BIC | Deviance | Δ*Dev* | LRT *df* | LRT *p* ($X^2$) |
|---|---|---|---|---|---|---|---|---|
| Full | 12 | -1875 | 3774.1 | 3833.6 | 3750.1 | 202.66 | 1 | < 2e-16 |
| Restricted | 11 | -1976 | 3974.7 | 4029.3 | 3952.7 | | | |
| **Random effects** | | | **Obs** | | **Variance** | | **Std** | |
| Bird ID | | | 324 | | 0.03551 | | 0.1885 | |
| Weekend | | | 21 | | 0.07231 | | 0.2689 | |
| Logger | | | 8 | | 0.03916 | | 0.1979 | |
| **Fixed effects** | | | **Estimate** | | **SE** | | ***t*** | |
| Intercept | | | 7.6022 | | 0.19085 | | 39.83 | |
| Condition | | | -1.3756 | | 0.09185 | | -14.98 | |
| Age | | | -0.1115 | | 0.12201 | | -0.91 | |
| Species | | | -0.0681 | | 0.09791 | | -0.70 | |
| Season | | | -0.0333 | | 0.20173 | | -0.17 | |
| Time | | | -0.0982 | | 0.05100 | | -1.93 | |
| N birds | | | 0.0141 | | 0.05324 | | 0.26 | |
| Attack | | | -0.0107 | | 0.01794 | | -0.60 | |

Generalized linear mixed effect model with *Logit* link and binomial error term. Number of observations: 1054. Estimates based on maximum likelihood approximation. Δ*Dev:* deviance difference between the full model and the restricted model without condition , LRT: likelihood ratio test, df: degrees of freedom, Std: standard deviation. BirdID: individual bird identity, Weekend: experimental weekend (replicate), Logger: location, Condition: attack vs. control, Age: adult vs. young, Species: great tit vs blue tit, Season: experimental season 2012/13 vs. 2013/14, Time: time of the day, attack: number of attacks previously experienced by the bird.

**Supplementary Table 3f:** Time to return: Attack vs. post-attack matched control.

| Model | *df* | *log* link | AIC | BIC | Deviance | Δ*Dev* | LRT *df* | LRT *p* ($X^2$) |
|---|---|---|---|---|---|---|---|---|
| Full | 12 | -1760 | 3544.5 | 3603.5 | 3520.5 | 132.9 | 1 | < 2e-16 |
| Restricted | 11 | -1826 | 3675.4 | 3729.5 | 3653.4 | | | |
| **Random effects** | | | **Obs** | | **Variance** | | **Std** | |
| Bird ID | | | 305 | | 0.01548 | | 0.1244 | |
| Weekend | | | 21 | | 0.03743 | | 0.1935 | |
| Logger | | | 8 | | 0.02403 | | 0.1550 | |
| **Fixed effects** | | | **Estimate** | | **SE** | | ***t*** | |
| Intercept | | | 7.35842 | | 0.15795 | | 46.59 | |
| Condition | | | -1.10413 | | 0.09103 | | -12.13 | |
| Age | | | 0.05083 | | 0.11333 | | 0.45 | |
| Species | | | -0.08846 | | 0.09490 | | -0.93 | |
| Season | | | -0.09741 | | 0.16238 | | -0.60 | |
| Time | | | -0.03163 | | 0.04739 | | -0.67 | |
| N birds | | | 0.00134 | | 0.05104 | | 0.03 | |
| Attack | | | 0.04009 | | 0.01660 | | 2.42 | |

Generalized linear mixed effect model with *Logit* link and binomial error term. Number of observations: 1011. Estimates based on maximum likelihood approximation. Δ*Dev:* deviance difference between the full model and the restricted model without condition , LRT: likelihood ratio test, df: degrees of freedom, Std: standard deviation. BirdID: individual bird identity, Weekend: experimental weekend (replicate), Logger: location, Condition: attack vs. control, Age: adult vs. young, Species: great tit vs blue tit, Season: experimental season 2012/13 vs. 2013/14, Time: time of the day, attack: number of attacks previously experienced by the bird.

**Supplementary Table 4a: Experimental releases**

| Release | Location | Date | Release time | Start GE | End GE | Birds | Follow-up |
|---|---|---|---|---|---|---|---|
| 1 | 1a | Sun 3 Feb 2013 | 12:09:57 | 12:08:21 | 12:10:00 | 12 | Y |
| 2 | 1a | Sun 3 Feb 2013 | 13:07:41 | 13:03:32 | 13:07:44 | 8 | Y |
| 3 | 3b | Sun 3 Feb 2013 | 9:48:01 | 9:46:20 | 9:48:17 | 2 | Y |
| 4 | 3b | Sun 3 Feb 2013 | 12:16:53 | 12:16:52 | 12:16:56 | 1 | Y |
| 5 | 4b | Sun 3 Feb 2013 | 10:07:05 | 10:03:57 | 10:07:10 | 4 | Y |
| 6 | 1a | Sat 9 Feb 2013 | 10:21:06 | 10:16:03 | 10:21:11 | 11 | Y |
| 7 | 3b | Sat 9 Feb 2013 | 11:25:13 | 11:22:22 | 11:26:22 | 4 | Y |
| 8 | 3b | Sat 9 Feb 2013 | 16:12:58 | 16:08:12 | 16:16:44 | 2 | Y |
| 9 | 3h | Sat 9 Feb 2013 | 10:02:39 | 10:02:36 | 10:02:42 | 1 | Y |
| 10 | 4b | Sat 9 Feb 2013 | 9:11:42 | 9:08:44 | 9:11:45 | 7 | Y |
| 11 | 4b | Sat 9 Feb 2013 | 12:10:58 | 12:07:15 | 12:11:01 | 6 | Y |
| 12 | 6f | Sat 9 Feb 2013 | 9:05:50 | 9:01:51 | 9:05:54 | 10 | Y |
| 13 | 7h | Sat 9 Feb 2013 | 9:55:38 | 9:51:48 | 9:55:39 | 18 | Y |
| 14 | 3h | Sat 16 Feb 2013 | 10:08:26 | 10:08:25 | 10:19:01 | 2 | Y |
| 15 | 4b | Sat 16 Feb 2013 | 9:05:11 | 9:02:06 | 9:05:14 | 8 | Y |
| 16 | 4b | Sat 16 Feb 2013 | 11:37:34 | 11:29:49 | 11:37:38 | 9 | Y |
| 17 | 6f | Sat 16 Feb 2013 | 9:36:36 | 9:32:30 | 9:36:39 | 5 | Y |
| 18 | 6f | Sat 16 Feb 2013 | 13:32:18 | 13:30:55 | 13:32:21 | 3 | Y |
| 19 | 7h | Sat 16 Feb 2013 | 11:21:55 | 11:18:36 | 11:21:59 | 10 | Y |
| 20 | 1a | Sat 23 Feb 2013 | 9:24:21 | 9:20:58 | 9:24:25 | 4 | Y |
| 21 | 3h | Sat 23 Feb 2013 | 11:42:40 | 11:40:57 | 11:42:44 | 2 | Y |
| 22 | 6f | Sat 23 Feb 2013 | 9:17:28 | 9:13:03 | 9:17:31 | 6 | Y |
| 23 | 6f | Sat 23 Feb 2013 | 12:36:00 | 12:31:58 | 12:36:04 | 19 | Y |
| 24 | 6f | Sat 23 Feb 2013 | 14:30:39 | 14:26:14 | 14:30:43 | 9 | Y |
| 25 | 7h | Sat 23 Feb 2013 | 9:10:08 | 9:03:32 | 9:10:13 | 13 | Y |
| 26 | 7h | Sat 23 Feb 2013 | 12:57:55 | 12:54:43 | 12:57:59 | 12 | Y |
| 27 | 1a | Sat 2 Mar 2013 | 10:44:04 | 10:38:50 | 10:44:07 | 10 | Y |
| 28 | 1a | Sat 2 Mar 2013 | 14:31:59 | 14:28:56 | 14:32:02 | 9 | Y |
| 29 | 1a | Sat 2 Mar 2013 | 15:27:59 | 15:22:33 | 15:28:02 | 13 | Y |
| 30 | 6f | Sat 2 Mar 2013 | 11:15:40 | 11:09:43 | 11:15:45 | 11 | Y |
| 31 | 7h | Sat 2 Mar 2013 | 11:47:23 | 11:46:50 | 11:47:27 | 4 | Y |
| 32 | 4b | Sun 3 Mar 2013 | 11:25:13 | 11:22:35 | 11:25:16 | 6 | Y |
| 33 | 4b | Sun 3 Mar 2013 | 15:33:27 | 15:29:36 | 15:33:30 | 5 | Y |
| 34 | 1c | Sat 9 Nov 2013 | 10:07:42 | 10:06:09 | 10:07:45 | 2 | Y |
| 35 | 1c | Sat 9 Nov 2013 | 15:09:39 | 15:05:58 | 15:09:42 | 9 | Y |
| 36 | 1c | Sat 16 Nov 2013 | 9:37:35 | 9:34:02 | 9:37:40 | 9 | Y |
| 37 | 1c | Sat 16 Nov 2013 | 12:46:04 | 12:39:00 | 12:46:08 | 12 | Y |
| 38 | 3h | Sat 23 Nov 2013 | 9:37:02 | 9:35:03 | 9:37:07 | 2 | Y |
| 39 | 1c | Sat 30 Nov 2013 | 9:12:23 | 9:09:11 | 9:13:53 | 5 | Y |
| 40 | 1c | Sat 30 Nov 2013 | 11:23:33 | 11:18:35 | 11:23:37 | 6 | Y |
| 41 | 1c | Sat 30 Nov 2013 | 13:33:01 | 13:28:56 | 13:33:04 | 11 | Y |

**Supplementary Table 4a cont.**

| | | | | | | | |
|---|---|---|---|---|---|---|---|
| 42 | 1c | Sat 30 Nov 2013 | 15:26:37 | 15:24:29 | 15:26:40 | 8 | Y |
| 43 | 1c | Sat 7 Dec 2013 | 13:16:46 | 13:11:10 | 13:16:50 | 16 | Y |
| 44 | 1c | Sat 7 Dec 2013 | 15:06:50 | 15:03:32 | 15:06:53 | 6 | Y |
| 45 | 3g | Sat 7 Dec 2013 | 8:47:37 | 8:45:32 | 8:47:40 | 2 | Y |
| 46 | 3g | Sat 7 Dec 2013 | 12:45:31 | 12:44:01 | 12:45:34 | 1 | Y |
| 47 | 3h | Sat 7 Dec 2013 | 14:14:38 | 14:11:33 | 14:14:42 | 7 | Y |
| 48 | 4b | Sat 7 Dec 2013 | 14:15:50 | 14:13:34 | 14:15:55 | 3 | Y |
| 49 | 1c | Sat 14 Dec 2013 | 9:38:56 | 9:37:10 | 9:39:34 | 4 | Y |
| 50 | 1c | Sat 14 Dec 2013 | 12:53:46 | 12:49:49 | 12:53:49 | 8 | Y |
| 51 | 3g | Sat 14 Dec 2013 | 9:47:46 | 9:45:45 | 9:49:29 | 3 | Y |
| 52 | 3g | Sat 14 Dec 2013 | 12:20:03 | 12:20:02 | 12:23:26 | 2 | Y |
| 53 | 3g | Sat 14 Dec 2013 | 13:11:46 | 13:04:05 | 13:11:49 | 4 | Y |
| 54 | 3h | Sat 14 Dec 2013 | 9:08:13 | 9:08:13 | 9:08:17 | 1 | Y |
| 55 | 3h | Sat 14 Dec 2013 | 12:41:09 | 12:38:07 | 12:41:13 | 8 | Y |
| 56 | 1c | Sat 21 Dec 2013 | 12:52:56 | 12:51:53 | 12:54:24 | 12 | Y |
| 57 | 3g | Sat 21 Dec 2013 | 9:40:04 | 9:40:04 | 9:40:08 | 1 | Y |
| 58 | 3g | Sat 21 Dec 2013 | 12:11:57 | 12:11:26 | 12:15:00 | 2 | Y |
| 59 | 4b | Sat 21 Dec 2013 | 9:31:01 | 9:28:54 | 9:31:06 | 2 | Y |
| 60 | 1c | Sat 4 Jan 2014 | 9:54:39 | 9:51:50 | 9:56:17 | 13 | Y |
| 61 | 1c | Sat 4 Jan 2014 | 12:10:49 | 12:08:39 | 12:10:53 | 7 | Y |
| 62 | 1c | Sat 4 Jan 2014 | 13:00:04 | 12:55:44 | 13:00:08 | 7 | Y |
| 63 | 1c | Sat 4 Jan 2014 | 15:41:43 | 15:40:57 | 15:41:47 | 2 | Y |
| 64 | 6f | Sat 4 Jan 2014 | 12:40:58 | 12:37:31 | 12:41:02 | 1 | Y |
| 65 | 1c | Sat 11 Jan 2014 | 9:51:37 | 9:47:42 | 9:51:41 | 12 | Y |
| 66 | 1c | Sat 11 Jan 2014 | 12:18:43 | 12:16:45 | 12:18:47 | 4 | Y |
| 67 | 1c | Sat 11 Jan 2014 | 14:11:03 | 14:08:51 | 14:11:07 | 4 | Y |
| 68 | 3g | Sat 11 Jan 2014 | 9:37:49 | 9:36:30 | 9:40:08 | 3 | Y |
| 69 | 3g | Sat 11 Jan 2014 | 13:17:48 | 13:14:07 | 13:17:52 | 4 | Y |
| 70 | 3g | Sat 11 Jan 2014 | 14:34:32 | 14:31:50 | 14:34:35 | 4 | Y |
| 71 | 3g | Sat 11 Jan 2014 | 15:45:40 | 15:42:48 | 15:45:43 | 6 | Y |
| 72 | 4b | Sat 11 Jan 2014 | 10:33:33 | 10:30:04 | 10:33:36 | 3 | Y |
| 73 | 4b | Sat 11 Jan 2014 | 14:18:47 | 14:15:36 | 14:18:51 | 5 | Y |
| 74 | 6f | Sat 11 Jan 2014 | 11:19:15 | 11:16:08 | 11:19:20 | 3 | Y |
| 75 | 1c | Sat 18 Jan 2014 | 11:43:31 | 11:40:12 | 11:43:34 | 12 | Y |
| 76 | 1c | Sat 18 Jan 2014 | 13:24:19 | 13:22:43 | 13:24:21 | 9 | Y |
| 77 | 1c | Sat 18 Jan 2014 | 15:15:16 | 15:11:34 | 15:15:30 | 10 | Y |
| 78 | 3g | Sat 18 Jan 2014 | 10:59:31 | 10:56:53 | 10:59:34 | 8 | Y |
| 79 | 4b | Sat 18 Jan 2014 | 10:08:20 | 10:03:43 | 10:08:25 | 4 | Y |
| 80 | 6f | Sat 18 Jan 2014 | 11:54:15 | 11:49:46 | 11:54:19 | 6 | Y |
| 81 | 1c | Sat 25 Jan 2014 | 11:30:40 | 11:27:40 | 11:30:43 | 6 | Y |
| 82 | 3g | Sat 25 Jan 2014 | 10:36:31 | 10:35:20 | 10:36:34 | 2 | Y |
| 83 | 4b | Sat 25 Jan 2014 | 9:54:35 | 9:49:40 | 9:54:39 | 6 | Y |
| 84 | 6f | Sat 25 Jan 2014 | 10:30:21 | 10:25:50 | 10:30:25 | 9 | Y |
| 85 | 1c | Sat 1 Feb 2014 | 9:57:16 | 9:51:59 | 9:57:19 | 5 | Y |

**Supplementary Table 4a cont.**

| | | | | | | | |
|---|---|---|---|---|---|---|---|
| 86 | 1c | Sat 1 Feb 2014 | 13:05:38 | 13:00:19 | 13:06:18 | 9 | Y |
| 87 | 1c | Sat 1 Feb 2014 | 13:33:57 | 13:30:46 | 13:34:00 | 8 | Y |
| 89 | 3h | Sat 1 Feb 2014 | 8:27:49 | 8:24:46 | 8:27:53 | 5 | Y |
| 90 | 6f | Sat 1 Feb 2014 | 11:06:44 | 11:01:43 | 11:07:36 | 9 | Y |
| 91 | 1c | Sat 8 Feb 2014 | 7:48:06 | 7:46:46 | 7:48:09 | 3 | Y |
| 92 | 3g | Sat 8 Feb 2014 | 8:44:49 | 8:41:49 | 8:45:03 | 3 | Y |
| 93 | 3g | Sat 8 Feb 2014 | 11:39:40 | 11:36:09 | 11:39:44 | 7 | Y |
| 94 | 4b | Sat 8 Feb 2014 | 8:13:08 | 8:03:44 | 8:13:11 | 6 | Y |
| 95 | 6f | Sat 8 Feb 2014 | 9:29:12 | 9:27:53 | 9:29:15 | 2 | Y |
| 96 | 6f | Sat 8 Feb 2014 | 12:30:19 | 12:27:49 | 12:30:23 | 11 | Y |
| 97 | 4b | Sat 15 Feb 2014 | 8:09:47 | 8:08:17 | 8:09:51 | 2 | Y |
| 98 | 4b | Sat 15 Feb 2014 | 9:43:56 | 9:40:45 | 9:44:00 | 3 | Y |
| 99 | 4b | Sat 15 Feb 2014 | 10:18:25 | 10:14:14 | 10:18:28 | 5 | Y |
| 100 | 6f | Sat 15 Feb 2014 | 9:41:57 | 9:38:30 | 9:42:01 | 7 | Y |
| 101 | 6f | Sat 15 Feb 2014 | 9:53:51 | 9:52:28 | 9:53:53 | 4 | Y |
| 102 | 6f | Sat 15 Feb 2014 | 10:06:20 | 10:03:39 | 10:06:24 | 10 | Y |
| 103 | 4b | Sat 22 Feb 2014 | 9:25:48 | 9:23:23 | 9:25:51 | 5 | Y |
| 104 | 4b | Sat 22 Feb 2014 | 9:40:31 | 9:34:39 | 9:40:35 | 8 | Y |
| 105 | 4b | Sat 22 Feb 2014 | 12:14:37 | 12:08:35 | 12:14:41 | 7 | Y |
| 106 | 6f | Sat 22 Feb 2014 | 13:15:27 | 13:14:08 | 13:15:30 | 3 | Y |
| 107 | 1c | Sat 1 Mar 2014 | 7:36:09 | 7:33:55 | 7:36:13 | 8 | Y |
| 108 | 1c | Sat 1 Mar 2014 | 8:03:39 | 8:02:06 | 8:03:42 | 3 | Y |
| 109 | 1c | Sat 1 Mar 2014 | 9:14:52 | 9:10:55 | 9:14:54 | 7 | Y |
| 110 | 1c | Sat 1 Mar 2014 | 11:33:13 | 11:31:11 | 11:33:15 | 3 | N |
| 111 | 4b | Sat 1 Mar 2014 | 11:14:43 | 11:11:38 | 11:14:47 | 3 | Y |
| 112 | 4b | Sat 1 Mar 2014 | 11:53:30 | 11:52:37 | 11:53:37 | 5 | Y |

Release: consecutive release number, Location: name of the logger, GE: gathering event, Birds: number of birds present at the gathering event, Follow-up: whether birds returned to the feeder after the GE (Y: yes, N: no)

**Supplementary Table 4b: Post-treatment control**

| Rel. | Date matched control | Start GE | End GE | Birds | Follow-up | Date gap-matched control | Start GE | End GE | Birds | Follow-up |
|---|---|---|---|---|---|---|---|---|---|---|
| 1 | Mon 11 Feb 2013 | 13:56:17 | 13:58:04 | 1 | Y | Mon 11 Feb 2013 | 7:49:07 | 7:49:15 | 1 | Y |
| 2 | Mon 11 Feb 2013 | 13:56:17 | 13:58:04 | 1 | Y | Mon 11 Feb 2013 | 8:00:39 | 8:05:24 | 5 | Y |
| 3 | Mon 11 Feb 2013 | 9:48:54 | 9:50:27 | 4 | Y | Mon 11 Feb 2013 | 13:38:03 | 13:42:07 | 3 | Y |
| 4 | Mon 11 Feb 2013 | 12:22:38 | 12:22:43 | 1 | Y | Mon 11 Feb 2013 | 14:59:15 | 15:02:53 | 3 | Y |
| 5 | Mon 11 Feb 2013 | 10:11:54 | 10:14:10 | 5 | Y | Mon 11 Feb 2013 | 7:35:13 | 7:35:54 | 2 | Y |
| 6 | Sun 10 Feb 2013 | 7:52:04 | 7:52:15 | 1 | Y | Sun 10 Feb 2013 | 7:54:45 | 7:55:52 | 2 | Y |
| 7 | Sun 10 Feb 2013 | 11:38:38 | 11:39:50 | 1 | Y | Sun 10 Feb 2013 | 13:57:49 | 14:02:08 | 6 | Y |
| 8 | Sun 10 Feb 2013 | 15:38:58 | 15:45:08 | 2 | Y | | | | | |
| 9 | Sun 10 Feb 2013 | 16:14:01 | 16:19:49 | 2 | Y | Sun 10 Feb 2013 | 11:25:05 | 11:31:23 | 10 | Y |
| 10 | Sun 10 Feb 2013 | 10:19:02 | 10:19:15 | 2 | Y | Sun 10 Feb 2013 | 10:52:55 | 10:58:42 | 10 | Y |

**Supplementary Table 4b cont.**

| | | | | | | | | | | |
|---|---|---|---|---|---|---|---|---|---|---|
| 11 | Sun 10 Feb 2013 | 9:15:07 | 9:20:57 | 5 | Y | Sun 10 Feb 2013 | 13:27:02 | 13:32:50 | 15 | Y |
| 12 | Sun 10 Feb 2013 | 12:11:27 | 12:15:36 | 7 | Y | Sun 10 Feb 2013 | 8:50:37 | 8:58:13 | 8 | Y |
| 13 | Sun 10 Feb 2013 | 9:08:27 | 9:09:34 | 2 | Y | Sun 10 Feb 2013 | 9:55:41 | 10:01:34 | 12 | Y |
| 14 | Sun 10 Feb 2013 | 9:55:39 | 9:58:55 | 2 | Y | Sun 17 Feb 2013 | 16:20:25 | 16:23:50 | 9 | Y |
| 15 | Sun 17 Feb 2013 | 8:53:39 | 8:57:13 | 4 | Y | Sun 17 Feb 2013 | 15:15:41 | 15:21:03 | 8 | Y |
| 16 | Sun 17 Feb 2013 | 12:30:45 | 12:32:22 | 2 | Y | Sun 17 Feb 2013 | 13:11:12 | 13:16:20 | 7 | Y |
| 17 | Sun 17 Feb 2013 | 10:13:40 | 10:13:45 | 1 | Y | Sun 17 Feb 2013 | 12:07:03 | 12:09:38 | 2 | Y |
| 18 | Sun 17 Feb 2013 | 9:06:52 | 9:10:23 | 10 | Y | Sun 17 Feb 2013 | 11:14:36 | 11:22:17 | 14 | Y |
| 19 | Sun 17 Feb 2013 | 11:42:45 | 11:44:45 | 10 | Y | Sun 17 Feb 2013 | 8:41:45 | 8:51:07 | 10 | Y |
| 20 | Sun 17 Feb 2013 | 9:39:13 | 9:43:26 | 4 | Y | Sun 24 Feb 2013 | 11:38:10 | 11:38:29 | 3 | Y |
| 21 | Sun 17 Feb 2013 | 13:41:46 | 13:45:38 | 4 | Y | | | | | |
| 22 | Sun 17 Feb 2013 | 11:24:33 | 11:27:37 | 11 | Y | Sun 24 Feb 2013 | 14:39:52 | 14:41:59 | 5 | Y |
| 23 | Sun 24 Feb 2013 | 9:28:32 | 9:33:18 | 4 | Y | Sun 24 Feb 2013 | 10:40:54 | 10:45:39 | 6 | Y |
| 24 | Sun 24 Feb 2013 | 14:34:47 | 14:38:34 | 1 | Y | Sun 24 Feb 2013 | 15:57:20 | 16:07:32 | 39 | Y |
| 25 | Sun 24 Feb 2013 | 9:20:13 | 9:22:30 | 2 | Y | Sun 24 Feb 2013 | 7:15:12 | 7:18:28 | 3 | Y |
| 26 | Sun 24 Feb 2013 | 12:37:44 | 12:40:29 | 6 | Y | Sun 24 Feb 2013 | 12:03:32 | 12:07:02 | 7 | Y |
| 27 | Sun 24 Feb 2013 | 14:41:05 | 14:41:12 | 1 | Y | Sun 3 Mar 2013 | 16:51:12 | 16:55:50 | 5 | Y |
| 28 | Sun 24 Feb 2013 | 9:11:55 | 9:14:49 | 12 | Y | Sun 3 Mar 2013 | 16:03:44 | 16:09:34 | 12 | Y |
| 29 | Sun 24 Feb 2013 | 13:00:37 | 13:07:22 | 11 | Y | Sun 3 Mar 2013 | 7:06:55 | 7:09:10 | 2 | Y |
| 30 | Sun 3 Mar 2013 | 10:53:13 | 10:53:20 | 1 | Y | Sun 3 Mar 2013 | 13:00:42 | 13:06:02 | 9 | Y |
| 31 | Sun 3 Mar 2013 | 14:34:41 | 14:37:52 | 6 | Y | Sun 3 Mar 2013 | 16:31:03 | 16:34:36 | 4 | Y |
| 32 | Sun 3 Mar 2013 | 15:35:34 | 15:38:34 | 8 | Y | Sat 2 Mar 2013 | 9:10:05 | 9:15:56 | 7 | Y |
| 33 | Sun 3 Mar 2013 | 11:23:32 | 11:30:00 | 6 | Y | Sat 2 Mar 2013 | 7:57:29 | 8:01:05 | 5 | Y |
| 34 | Sun 3 Mar 2013 | 11:52:36 | 11:57:14 | 6 | Y | Sun 10 Nov 2013 | 12:10:29 | 12:17:03 | 4 | Y |
| 35 | Sat 2 Mar 2013 | 7:58:40 | 8:00:47 | 6 | Y | Sun 10 Nov 2013 | 9:23:30 | 9:27:32 | 4 | Y |
| 36 | Sat 2 Mar 2013 | 7:58:40 | 8:00:47 | 6 | Y | Sun 17 Nov 2013 | 14:59:18 | 15:03:55 | 3 | Y |
| 37 | Sun 10 Nov 2013 | 10:11:18 | 10:11:30 | 2 | Y | Sun 17 Nov 2013 | 11:48:18 | 11:54:49 | 6 | Y |
| 38 | Sun 10 Nov 2013 | 15:15:38 | 15:19:31 | 7 | Y | Sun 24 Nov 2013 | 9:38:23 | 9:45:47 | 8 | Y |
| 39 | Sun 17 Nov 2013 | 9:40:48 | 9:44:48 | 4 | Y | Sun 8 Dec 2013 | 8:35:58 | 8:41:33 | 3 | Y |
| 40 | Sun 17 Nov 2013 | 12:50:46 | 12:53:43 | 6 | Y | Sun 8 Dec 2013 | 11:25:15 | 11:28:11 | 1 | Y |
| 41 | Sun 24 Nov 2013 | 9:39:06 | 9:41:26 | 4 | Y | Sun 8 Dec 2013 | 15:21:17 | 15:22:19 | 2 | Y |
| 42 | Sun 8 Dec 2013 | 9:22:42 | 9:29:00 | 10 | Y | Sun 8 Dec 2013 | 11:51:37 | 11:55:47 | 4 | Y |
| 43 | Sun 8 Dec 2013 | 11:26:53 | 11:30:56 | 8 | Y | Sun 8 Dec 2013 | 11:53:32 | 11:59:04 | 3 | Y |
| 44 | Sun 8 Dec 2013 | 13:42:22 | 13:46:57 | 9 | Y | Sun 8 Dec 2013 | 11:28:08 | 11:32:22 | 6 | Y |
| 45 | Sun 8 Dec 2013 | 15:27:28 | 15:28:31 | 5 | Y | Sun 8 Dec 2013 | 13:14:51 | 13:21:03 | 6 | Y |
| 46 | Sun 8 Dec 2013 | 9:54:44 | 9:58:34 | 4 | Y | Sun 8 Dec 2013 | 10:04:44 | 10:07:55 | 3 | Y |
| 47 | Sun 8 Dec 2013 | 13:30:05 | 13:31:13 | 2 | Y | Sun 8 Dec 2013 | 11:56:12 | 12:00:04 | 10 | Y |
| 48 | Sun 8 Dec 2013 | 15:16:43 | 15:19:44 | 6 | Y | Sun 8 Dec 2013 | 13:14:51 | 13:21:03 | 6 | Y |
| 49 | Sun 8 Dec 2013 | 8:47:55 | 8:47:59 | 1 | Y | Sun 15 Dec 2013 | 8:15:31 | 8:18:04 | 4 | Y |
| 50 | Sun 8 Dec 2013 | 13:08:57 | 13:10:46 | 1 | Y | Sun 15 Dec 2013 | 11:01:18 | 11:08:28 | 11 | Y |
| 51 | Sun 8 Dec 2013 | 15:33:28 | 15:39:50 | 1 | Y | Sun 22 Dec 2013 | 9:44:33 | 9:49:11 | 4 | Y |
| 52 | Sun 8 Dec 2013 | 14:18:45 | 14:21:19 | 4 | Y | Sun 22 Dec 2013 | 9:44:33 | 9:49:11 | 4 | Y |
| 53 | Sun 15 Dec 2013 | 9:39:01 | 9:41:49 | 6 | Y | Sun 22 Dec 2013 | 11:20:30 | 11:22:20 | 3 | Y |

**Supplementary Table 4b cont.**

| | | | | | | | | | | |
|---|---|---|---|---|---|---|---|---|---|---|
| 54 | Sun 15 Dec 2013 | 12:53:53 | 12:58:57 | 7 | Y | Sun 15 Dec 2013 | 9:01:10 | 9:10:30 | 4 | Y |
| 55 | Sun 22 Dec 2013 | 9:52:23 | 9:52:27 | 1 | Y | Sun 15 Dec 2013 | 9:01:10 | 9:10:30 | 4 | Y |
| 56 | Sun 22 Dec 2013 | 12:45:26 | 12:45:31 | 1 | Y | Sun 22 Dec 2013 | 10:14:21 | 10:17:45 | 4 | Y |
| 57 | Sun 22 Dec 2013 | 13:12:09 | 13:16:32 | 1 | Y | Sun 22 Dec 2013 | 13:27:46 | 13:34:26 | 12 | Y |
| 58 | Sun 15 Dec 2013 | 9:25:56 | 9:28:57 | 3 | Y | Sun 22 Dec 2013 | 9:44:33 | 9:49:11 | 4 | Y |
| 59 | Sun 15 Dec 2013 | 8:21:14 | 8:21:18 | 1 | Y | Sun 22 Dec 2013 | 12:59:52 | 13:04:02 | 4 | Y |
| 60 | Sun 22 Dec 2013 | 12:58:11 | 13:01:46 | 6 | Y | Sun 5 Jan 2014 | 14:42:50 | 14:48:28 | 5 | Y |
| 61 | Sun 22 Dec 2013 | 9:45:27 | 9:46:28 | 1 | Y | Sun 5 Jan 2014 | 14:42:50 | 14:48:28 | 5 | Y |
| 62 | Sun 22 Dec 2013 | 12:18:10 | 12:18:14 | 1 | Y | Sun 5 Jan 2014 | 13:01:56 | 13:06:31 | 7 | Y |
| 63 | Sun 22 Dec 2013 | 9:39:28 | 9:46:35 | 3 | Y | | | | | |
| 64 | Sun 5 Jan 2014 | 9:56:15 | 9:58:24 | 4 | Y | Sun 5 Jan 2014 | 8:47:47 | 8:53:14 | 6 | Y |
| 65 | Sun 5 Jan 2014 | 12:13:11 | 12:15:43 | 7 | Y | Sun 12 Jan 2014 | 13:56:29 | 14:00:18 | 6 | Y |
| 66 | Sun 5 Jan 2014 | 13:01:45 | 13:05:29 | 16 | Y | Sun 12 Jan 2014 | 9:55:45 | 10:02:44 | 6 | Y |
| 67 | Sun 5 Jan 2014 | 15:45:17 | 15:49:44 | 5 | Y | Sun 12 Jan 2014 | 15:17:38 | 15:24:17 | 15 | Y |
| 68 | Sun 5 Jan 2014 | 12:46:48 | 12:52:16 | 3 | Y | Sun 12 Jan 2014 | 10:14:37 | 10:19:37 | 4 | Y |
| 69 | Sun 12 Jan 2014 | 9:54:52 | 9:56:44 | 1 | Y | Sun 12 Jan 2014 | 13:15:14 | 13:21:36 | 19 | Y |
| 70 | Sun 12 Jan 2014 | 12:19:50 | 12:26:07 | 14 | Y | Sun 12 Jan 2014 | 11:34:01 | 11:39:36 | 13 | Y |
| 71 | Sun 12 Jan 2014 | 14:14:47 | 14:14:48 | 1 | Y | Sun 12 Jan 2014 | 11:49:50 | 11:54:00 | 10 | Y |
| 72 | Sun 12 Jan 2014 | 9:40:21 | 9:40:24 | 1 | Y | Sun 12 Jan 2014 | 7:59:37 | 8:04:24 | 4 | Y |
| 73 | Sun 12 Jan 2014 | 13:24:09 | 13:26:55 | 6 | Y | Sun 12 Jan 2014 | 9:33:54 | 9:40:44 | 11 | Y |
| 74 | Sun 12 Jan 2014 | 14:39:27 | 14:44:21 | 8 | Y | Sun 12 Jan 2014 | 13:21:54 | 13:27:31 | 14 | Y |
| 75 | Sun 12 Jan 2014 | 15:47:53 | 15:52:06 | 6 | Y | Sun 19 Jan 2014 | 15:05:54 | 15:14:11 | 8 | Y |
| 76 | Sun 12 Jan 2014 | 10:37:06 | 10:38:45 | 3 | Y | Sun 19 Jan 2014 | 12:50:43 | 12:55:30 | 7 | Y |
| 77 | Sun 12 Jan 2014 | 14:36:17 | 15:55:24 | 3 | Y | Sun 19 Jan 2014 | 15:14:50 | 15:15:19 | 2 | Y |
| 78 | Sun 12 Jan 2014 | 11:21:30 | 11:27:26 | 8 | Y | Sun 19 Jan 2014 | 12:45:11 | 12:50:30 | 4 | Y |
| 79 | Sun 19 Jan 2014 | 11:46:13 | 11:48:46 | 2 | Y | Sun 19 Jan 2014 | 10:49:07 | 10:53:48 | 10 | Y |
| 80 | Sun 19 Jan 2014 | 13:26:44 | 13:34:02 | 11 | Y | Sun 19 Jan 2014 | 12:32:28 | 12:40:14 | 14 | Y |
| 81 | Sun 19 Jan 2014 | 15:33:42 | 15:34:34 | 6 | Y | Sun 26 Jan 2014 | 9:51:34 | 9:54:48 | 2 | Y |
| 82 | Sun 19 Jan 2014 | 11:04:07 | 11:09:14 | 5 | Y | Sun 26 Jan 2014 | 8:41:10 | 8:47:32 | 7 | Y |
| 83 | Sun 19 Jan 2014 | 10:08:24 | 10:13:22 | 10 | Y | Sun 26 Jan 2014 | 11:45:18 | 11:49:21 | 7 | Y |
| 84 | Sun 19 Jan 2014 | 12:02:25 | 12:04:54 | 4 | Y | Sun 26 Jan 2014 | 13:40:03 | 13:48:33 | 19 | Y |
| 85 | Sun 26 Jan 2014 | 11:39:47 | 11:44:04 | 6 | Y | Sun 2 Feb 2014 | 7:55:23 | 8:07:51 | 4 | Y |
| 86 | Sun 26 Jan 2014 | 10:41:52 | 10:45:42 | 3 | Y | Sun 2 Feb 2014 | 13:54:05 | 14:00:10 | 6 | Y |
| 87 | Sun 26 Jan 2014 | 10:05:24 | 10:06:54 | 2 | Y | Sun 2 Feb 2014 | 14:36:56 | 14:41:29 | 8 | Y |
| 88 | Sun 26 Jan 2014 | 10:34:16 | 10:39:03 | 10 | Y | Sun 2 Feb 2014 | 8:24:47 | 8:30:42 | 5 | Y |
| 89 | Sun 2 Feb 2014 | 9:59:49 | 10:00:14 | 2 | Y | Sun 2 Feb 2014 | 11:55:03 | 12:00:03 | 11 | Y |
| 90 | Sun 2 Feb 2014 | 13:07:44 | 13:09:31 | 2 | Y | Sun 2 Feb 2014 | 7:55:23 | 8:07:51 | 4 | Y |
| 91 | Sun 2 Feb 2014 | 13:33:58 | 13:37:04 | 6 | Y | Sun 9 Feb 2014 | 7:32:07 | 7:34:23 | 1 | Y |
| 92 | Sun 2 Feb 2014 | 8:45:29 | 8:45:33 | 1 | Y | Sun 9 Feb 2014 | 8:26:41 | 8:33:57 | 10 | Y |
| 93 | Sun 2 Feb 2014 | 8:47:02 | 8:47:06 | 1 | Y | Sun 9 Feb 2014 | 12:28:11 | 12:36:02 | 9 | Y |
| 94 | Sun 2 Feb 2014 | 11:07:32 | 11:10:13 | 5 | Y | Sun 9 Feb 2014 | 15:37:21 | 15:41:21 | 19 | Y |
| 95 | Sun 9 Feb 2014 | 8:08:37 | 8:12:36 | 4 | Y | Sun 9 Feb 2014 | 14:58:46 | 15:05:39 | 5 | Y |
| 96 | Sun 9 Feb 2014 | 8:48:07 | 8:56:49 | 1 | Y | Sun 9 Feb 2014 | 12:47:41 | 12:55:00 | 21 | Y |

**Supplementary Table 4b cont.**

| | | | | | | | | | | |
|---|---|---|---|---|---|---|---|---|---|---|
| 97 | Sun 9 Feb 2014 | 11:45:15 | 11:46:27 | 1 | Y | Sun 16 Feb 2014 | 15:29:06 | 15:35:14 | 12 | Y |
| 98 | Sun 9 Feb 2014 | 8:16:53 | 8:23:30 | 8 | Y | Sun 16 Feb 2014 | 16:08:10 | 16:16:07 | 11 | Y |
| 99 | Sun 9 Feb 2014 | 9:36:14 | 9:42:43 | 8 | Y | Sun 16 Feb 2014 | 15:20:57 | 15:26:26 | 10 | Y |
| 100 | Sun 9 Feb 2014 | 12:34:40 | 12:39:04 | 9 | Y | Sun 16 Feb 2014 | 8:05:12 | 8:09:36 | 11 | Y |
| 101 | Sun 16 Feb 2014 | 8:23:04 | 8:24:48 | 1 | Y | Sun 16 Feb 2014 | 8:05:12 | 8:09:36 | 11 | Y |
| 102 | Sun 16 Feb 2014 | 9:48:43 | 9:51:03 | 3 | Y | Sun 16 Feb 2014 | 8:44:57 | 8:51:54 | 8 | Y |
| 103 | Sun 16 Feb 2014 | 10:20:19 | 10:22:39 | 3 | Y | Sun 23 Feb 2014 | 12:24:23 | 12:29:56 | 9 | Y |
| 104 | Sun 16 Feb 2014 | 9:48:32 | 9:57:09 | 6 | Y | Sun 23 Feb 2014 | 14:15:10 | 14:21:37 | 13 | Y |
| 105 | Sun 16 Feb 2014 | 9:59:58 | 10:04:54 | 6 | Y | Sun 23 Feb 2014 | 11:27:17 | 11:35:43 | 15 | Y |
| 106 | Sun 16 Feb 2014 | 10:09:23 | 10:12:35 | 4 | Y | Sun 23 Feb 2014 | 10:44:57 | 10:48:16 | 4 | Y |
| 107 | Sun 23 Feb 2014 | 9:29:09 | 9:32:28 | 5 | Y | Sun 23 Feb 2014 | 7:59:58 | 8:06:15 | 8 | Y |
| 108 | Sun 23 Feb 2014 | 9:40:39 | 9:45:38 | 5 | Y | Sun 23 Feb 2014 | 8:13:28 | 8:18:38 | 5 | Y |
| 109 | Sun 23 Feb 2014 | 12:20:32 | 12:22:46 | 4 | Y | Sun 23 Feb 2014 | 16:35:43 | 16:41:09 | 4 | Y |
| 110 | Sun 23 Feb 2014 | 7:29:08 | 7:31:40 | 1 | Y | | | | | |
| 111 | Sun 23 Feb 2014 | 7:37:17 | 7:38:55 | 2 | Y | Sun 2 Mar 2014 | 15:40:52 | 15:47:23 | 11 | Y |
| 112 | Sun 23 Feb 2014 | 8:07:40 | 8:08:55 | 1 | Y | Sun 2 Mar 2014 | 8:30:18 | 8:36:47 | 4 | Y |

Rel.: consecutive release number, GE: gathering event, Birds: number of birds present at the gathering event, Follow-up: whether birds returned to the feeder after the gathering event (Y: yes, N: no)

**Supplementary Table 4c: Pre-treatment control**

| Rel. | Date matched control | Start GE | End GE | Birds | Follow-up | Date gap-matched control | Start GE | End GE | Birds | Follow-up |
|---|---|---|---|---|---|---|---|---|---|---|
| 1 | Sat 2 Feb 2013 | 12:09:58 | 12:10:27 | 4 | Y | Sat 2 Feb 2013 | 15:08:48 | 15:13:43 | 11 | Y |
| 2 | Sat 2 Feb 2013 | 13:10:46 | 13:11:35 | 2 | Y | Sat 2 Feb 2013 | 13:19:59 | 13:23:39 | 9 | Y |
| 3 | Sat 2 Feb 2013 | 10:00:50 | 10:00:54 | 1 | Y | Sat 2 Feb 2013 | 10:06:41 | 10:12:47 | 11 | Y |
| 4 | Sat 2 Feb 2013 | 12:22:05 | 12:28:23 | 3 | Y | Sat 2 Feb 2013 | 09:32:34 | 09:40:27 | 7 | Y |
| 5 | Sat 2 Feb 2013 | 10:08:14 | 10:13:27 | 3 | Y | Sat 2 Feb 2013 | 15:29:25 | 15:36:30 | 16 | Y |
| 6 | Fri 8 Feb 2013 | 10:22:03 | 10:29:10 | 5 | Y | Fri 8 Feb 2013 | 15:06:52 | 15:10:30 | 2 | Y |
| 7 | Fri 8 Feb 2013 | 11:39:34 | 11:40:13 | 1 | Y | Fri 8 Feb 2013 | 15:11:13 | 15:14:16 | 5 | Y |
| 8 | Fri 8 Feb 2013 | 15:42:39 | 15:46:57 | 3 | Y | Fri 8 Feb 2013 | 08:32:17 | 08:37:39 | 3 | Y |
| 9 | Fri 8 Feb 2013 | 16:24:21 | 16:26:22 | 1 | Y | | | | | |
| 10 | Fri 8 Feb 2013 | 10:06:06 | 10:08:10 | 2 | Y | Fri 8 Feb 2013 | 09:03:39 | 09:07:54 | 2 | Y |
| 11 | Fri 8 Feb 2013 | 09:14:19 | 09:16:51 | 2 | Y | Fri 8 Feb 2013 | 12:54:27 | 12:57:33 | 3 | Y |
| 12 | Fri 8 Feb 2013 | 12:15:05 | 12:23:01 | 6 | Y | Fri 8 Feb 2013 | 14:03:58 | 14:05:58 | 2 | Y |
| 13 | Fri 8 Feb 2013 | 09:08:15 | 09:08:54 | 1 | Y | Fri 8 Feb 2013 | 12:32:41 | 12:36:39 | 3 | Y |
| 14 | Fri 15 Feb 2013 | 10:01:21 | 10:02:38 | 6 | Y | Fri 15 Feb 2013 | 13:53:30 | 13:57:05 | 10 | Y |
| 15 | Fri 15 Feb 2013 | 08:55:07 | 08:56:07 | 2 | Y | Fri 15 Feb 2013 | 09:03:50 | 09:03:53 | 1 | Y |
| 16 | Fri 15 Feb 2013 | 12:28:50 | 12:31:40 | 3 | Y | Fri 15 Feb 2013 | 11:00:27 | 11:03:10 | 3 | Y |
| 17 | Fri 15 Feb 2013 | 10:15:51 | 10:19:46 | 1 | Y | Fri 15 Feb 2013 | 08:51:07 | 08:54:04 | 2 | Y |
| 18 | Fri 15 Feb 2013 | 09:06:12 | 09:08:44 | 2 | Y | Fri 15 Feb 2013 | 09:21:50 | 09:24:27 | 1 | Y |
| 19 | Fri 15 Feb 2013 | 11:41:59 | 11:42:50 | 3 | Y | Fri 15 Feb 2013 | 09:37:36 | 09:43:44 | 3 | Y |

**Supplementary Table 4c cont.**

| | | | | | | | | | | |
|---|---|---|---|---|---|---|---|---|---|---|
| 20 | Fri 15 Feb 2013 | 09:39:03 | 09:41:23 | 2 | Y | Fri 15 Feb 2013 | 14:35:01 | 14:41:50 | 6 | Y |
| 21 | Fri 15 Feb 2013 | 13:35:58 | 13:40:33 | 3 | Y | Fri 15 Feb 2013 | 07:36:40 | 07:43:09 | 5 | Y |
| 22 | Fri 15 Feb 2013 | 11:23:48 | 11:27:36 | 9 | Y | Fri 15 Feb 2013 | 13:53:30 | 13:57:05 | 10 | Y |
| 23 | Fri 22 Feb 2013 | 09:33:02 | 09:36:39 | 2 | Y | Fri 22 Feb 2013 | 14:41:08 | 14:41:41 | 3 | Y |
| 24 | Fri 1 Mar 2013 | 11:46:09 | 11:50:07 | 2 | Y | | | | | |
| 25 | Fri 22 Feb 2013 | 09:18:33 | 09:21:53 | 4 | Y | Fri 22 Feb 2013 | 10:44:09 | 10:47:48 | 3 | Y |
| 26 | Fri 22 Feb 2013 | 12:48:04 | 12:55:21 | 9 | Y | Fri 22 Feb 2013 | 07:14:30 | 07:21:06 | 3 | Y |
| 27 | Fri 22 Feb 2013 | 14:32:11 | 14:35:19 | 5 | Y | Fri 22 Feb 2013 | 07:14:30 | 07:21:06 | 3 | Y |
| 28 | Fri 22 Feb 2013 | 09:12:25 | 09:17:50 | 8 | Y | Fri 22 Feb 2013 | 07:24:10 | 07:24:14 | 1 | Y |
| 29 | Fri 22 Feb 2013 | 13:02:21 | 13:03:37 | 6 | Y | Fri 22 Feb 2013 | 16:00:19 | 16:03:48 | 2 | Y |
| 30 | Fri 1 Mar 2013 | 10:46:43 | 10:49:00 | 2 | Y | Fri 1 Mar 2013 | 11:35:01 | 11:39:40 | 6 | Y |
| 31 | Fri 1 Mar 2013 | 14:35:51 | 14:35:55 | 1 | Y | Fri 1 Mar 2013 | 15:28:15 | 15:31:58 | 5 | Y |
| 32 | Fri 1 Mar 2013 | 15:28:15 | 15:31:58 | 5 | Y | Fri 1 Mar 2013 | 09:59:58 | 10:04:11 | 3 | Y |
| 33 | Fri 1 Mar 2013 | 11:17:38 | 11:25:45 | 14 | Y | Fri 1 Mar 2013 | 16:29:42 | 16:34:03 | 7 | Y |
| 34 | Fri 1 Mar 2013 | 11:50:44 | 11:54:56 | 7 | Y | Fri 1 Mar 2013 | 07:11:35 | 07:15:15 | 2 | Y |
| 35 | Fri 15 Feb 2013 | 11:29:36 | 11:36:12 | 6 | Y | Fri 15 Feb 2013 | 08:51:07 | 08:54:04 | 2 | Y |
| 36 | Fri 15 Feb 2013 | 15:39:41 | 15:42:44 | 5 | Y | Fri 15 Feb 2013 | 08:51:07 | 08:54:04 | 2 | Y |
| 37 | Fri 8 Nov 2013 | 10:10:39 | 10:19:16 | 8 | Y | Fri 8 Nov 2013 | 07:34:25 | 07:34:31 | 1 | Y |
| 38 | Fri 8 Nov 2013 | 15:12:37 | 15:12:42 | 1 | Y | Fri 8 Nov 2013 | 08:05:57 | 08:09:44 | 4 | Y |
| 39 | Fri 15 Nov 2013 | 09:49:33 | 09:49:38 | 1 | Y | Fri 15 Nov 2013 | 07:29:42 | 07:35:12 | 10 | Y |
| 40 | Fri 15 Nov 2013 | 12:54:44 | 13:00:35 | 8 | Y | Fri 15 Nov 2013 | 09:06:55 | 09:08:46 | 1 | Y |
| 41 | Fri 22 Nov 2013 | 09:40:54 | 09:42:02 | 1 | Y | Fri 22 Nov 2013 | 11:31:12 | 11:36:28 | 7 | Y |
| 42 | Fri 29 Nov 2013 | 09:19:06 | 09:25:21 | 9 | Y | Fri 29 Nov 2013 | 09:48:48 | 09:50:50 | 4 | Y |
| 43 | Fri 29 Nov 2013 | 11:25:12 | 11:28:10 | 3 | Y | Fri 29 Nov 2013 | 13:36:08 | 13:42:41 | 13 | Y |
| 44 | Fri 29 Nov 2013 | 13:36:08 | 13:42:41 | 13 | Y | Fri 29 Nov 2013 | 08:19:01 | 08:23:12 | 3 | Y |
| 45 | Fri 29 Nov 2013 | 15:29:58 | 15:33:42 | 13 | Y | Fri 29 Nov 2013 | 14:41:25 | 14:45:41 | 14 | Y |
| 46 | Fri 6 Dec 2013 | 09:53:24 | 09:53:39 | 0 | Y | Fri 6 Dec 2013 | 14:42:03 | 14:42:58 | 0 | Y |
| 47 | Fri 6 Dec 2013 | 13:19:12 | 13:23:30 | 0 | Y | Fri 6 Dec 2013 | 07:59:58 | 08:00:19 | 0 | Y |
| 48 | Fri 6 Dec 2013 | 15:11:56 | 15:13:37 | 0 | Y | Fri 6 Dec 2013 | 14:32:29 | 14:36:58 | 0 | Y |
| 49 | Fri 6 Dec 2013 | 09:01:15 | 09:04:36 | 0 | Y | Fri 6 Dec 2013 | 09:53:56 | 09:58:21 | 0 | Y |
| 50 | Fri 6 Dec 2013 | 12:45:33 | 12:53:50 | 0 | Y | Fri 6 Dec 2013 | 09:06:38 | 09:11:41 | 0 | Y |
| 51 | Fri 6 Dec 2013 | 15:27:31 | 15:28:12 | 0 | Y | Fri 6 Dec 2013 | 13:30:06 | 13:33:39 | 0 | Y |
| 52 | Fri 6 Dec 2013 | 14:16:51 | 14:28:15 | 0 | Y | Fri 6 Dec 2013 | 08:21:26 | 08:29:40 | 0 | Y |
| 53 | Fri 13 Dec 2013 | 09:44:47 | 09:50:04 | 14 | Y | Fri 13 Dec 2013 | 08:28:49 | 08:34:53 | 12 | Y |
| 54 | Fri 13 Dec 2013 | 12:56:44 | 12:56:44 | 1 | Y | Fri 13 Dec 2013 | 14:05:46 | 14:09:44 | 9 | Y |
| 55 | Fri 13 Dec 2013 | 09:54:50 | 09:58:08 | 2 | Y | Fri 13 Dec 2013 | 12:16:53 | 12:21:26 | 5 | Y |
| 56 | Fri 13 Dec 2013 | 12:20:07 | 12:21:53 | 2 | Y | Fri 13 Dec 2013 | 09:55:41 | 10:00:55 | 10 | Y |
| 57 | Fri 13 Dec 2013 | 13:17:00 | 13:20:15 | 2 | Y | Fri 13 Dec 2013 | 11:41:15 | 11:47:45 | 4 | Y |
| 58 | Fri 13 Dec 2013 | 09:33:54 | 09:33:58 | 1 | Y | Fri 13 Dec 2013 | 14:08:12 | 14:11:14 | 2 | Y |
| 59 | Fri 13 Dec 2013 | 14:08:12 | 14:11:14 | 2 | Y | Fri 13 Dec 2013 | 11:13:55 | 11:21:37 | 11 | Y |
| 60 | Fri 20 Dec 2013 | 12:53:49 | 13:00:59 | 10 | Y | Fri 20 Dec 2013 | 09:57:39 | 09:58:20 | 2 | Y |
| 61 | Fri 20 Dec 2013 | 09:42:36 | 09:50:49 | 2 | Y | Fri 20 Dec 2013 | 12:30:44 | 12:34:22 | 7 | Y |
| 62 | Fri 20 Dec 2013 | 12:17:50 | 12:19:00 | 1 | Y | Fri 20 Dec 2013 | 08:40:34 | 08:41:28 | 1 | Y |

**Supplementary Table 4c cont.**

| | | | | | | | | | | |
|---|---|---|---|---|---|---|---|---|---|---|
| 63 | Fri 20 Dec 2013 | 09:37:56 | 09:38:00 | 1 | Y | Fri 20 Dec 2013 | 08:02:08 | 08:06:24 | 5 | Y |
| 64 | Fri 3 Jan 2014 | 09:56:26 | 09:58:59 | 6 | Y | Fri 3 Jan 2014 | 09:05:55 | 09:11:01 | 7 | Y |
| 65 | Fri 3 Jan 2014 | 12:22:44 | 12:27:51 | 18 | Y | Fri 3 Jan 2014 | 09:05:55 | 09:11:01 | 7 | Y |
| 66 | Fri 3 Jan 2014 | 13:04:19 | 13:06:11 | 12 | Y | Fri 3 Jan 2014 | 14:56:20 | 15:00:27 | 8 | Y |
| 67 | Fri 3 Jan 2014 | 15:44:29 | 15:50:33 | 12 | Y | | | | | |
| 68 | Fri 3 Jan 2014 | 12:59:12 | 13:04:30 | 6 | Y | Fri 3 Jan 2014 | 13:38:59 | 13:44:21 | 14 | Y |
| 69 | Fri 10 Jan 2014 | 09:55:54 | 09:56:46 | 5 | Y | Fri 10 Jan 2014 | 08:32:00 | 08:37:57 | 5 | Y |
| 70 | Fri 10 Jan 2014 | 12:23:34 | 12:24:44 | 6 | Y | Fri 10 Jan 2014 | 15:38:40 | 15:42:36 | 12 | Y |
| 71 | Fri 10 Jan 2014 | 14:14:17 | 14:19:23 | 25 | Y | Fri 10 Jan 2014 | 10:18:53 | 10:22:37 | 7 | Y |
| 72 | Fri 10 Jan 2014 | 09:43:26 | 09:43:30 | 1 | Y | Fri 10 Jan 2014 | 11:46:54 | 11:49:51 | 5 | Y |
| 73 | Fri 10 Jan 2014 | 13:19:15 | 13:24:03 | 2 | Y | Fri 10 Jan 2014 | 09:49:04 | 09:55:15 | 6 | Y |
| 74 | Fri 10 Jan 2014 | 14:36:49 | 14:39:43 | 3 | Y | Fri 10 Jan 2014 | 11:33:52 | 11:41:03 | 12 | Y |
| 75 | Fri 10 Jan 2014 | 15:46:35 | 15:50:03 | 5 | Y | Fri 10 Jan 2014 | 08:55:49 | 08:59:13 | 2 | Y |
| 76 | Fri 10 Jan 2014 | 10:38:54 | 10:41:47 | 2 | Y | Fri 10 Jan 2014 | 15:47:57 | 15:52:41 | 7 | Y |
| 77 | Fri 10 Jan 2014 | 14:21:46 | 14:25:01 | 8 | Y | Fri 10 Jan 2014 | 08:47:42 | 08:54:57 | 7 | Y |
| 78 | Fri 10 Jan 2014 | 11:22:53 | 11:28:15 | 7 | Y | Fri 10 Jan 2014 | 12:41:04 | 12:45:55 | 9 | Y |
| 79 | Fri 17 Jan 2014 | 11:44:42 | 11:46:30 | 4 | Y | Fri 17 Jan 2014 | 15:14:28 | 15:18:09 | 6 | Y |
| 80 | Fri 17 Jan 2014 | 13:24:38 | 13:24:44 | 2 | Y | Fri 17 Jan 2014 | 09:01:00 | 09:06:06 | 2 | Y |
| 81 | Fri 17 Jan 2014 | 15:34:00 | 15:39:30 | 23 | Y | Fri 17 Jan 2014 | 15:14:28 | 15:18:09 | 6 | Y |
| 82 | Fri 17 Jan 2014 | 11:03:32 | 11:06:18 | 4 | Y | Fri 17 Jan 2014 | 16:17:12 | 16:20:48 | 2 | Y |
| 83 | Fri 17 Jan 2014 | 10:14:22 | 10:16:15 | 3 | Y | Fri 17 Jan 2014 | 15:12:49 | 15:15:55 | 10 | Y |
| 84 | Fri 17 Jan 2014 | 11:54:28 | 11:56:44 | 5 | Y | Fri 17 Jan 2014 | 09:15:05 | 09:21:40 | 12 | Y |
| 85 | Fri 24 Jan 2014 | 11:32:57 | 11:36:53 | 7 | Y | Fri 24 Jan 2014 | 08:42:13 | 08:45:36 | 5 | Y |
| 86 | Fri 24 Jan 2014 | 10:38:45 | 10:39:42 | 1 | Y | Fri 24 Jan 2014 | 10:42:11 | 10:46:29 | 16 | Y |
| 87 | Fri 24 Jan 2014 | 09:56:27 | 09:59:44 | 2 | Y | Fri 24 Jan 2014 | 13:19:44 | 13:27:09 | 7 | Y |
| 88 | Fri 24 Jan 2014 | 10:31:02 | 10:31:07 | 1 | Y | Fri 24 Jan 2014 | 10:19:27 | 10:25:12 | 9 | Y |
| 89 | Fri 31 Jan 2014 | 09:59:20 | 09:59:47 | 5 | Y | Fri 31 Jan 2014 | 12:56:48 | 13:01:08 | 9 | Y |
| 90 | Fri 31 Jan 2014 | 13:07:37 | 13:11:01 | 7 | Y | Fri 31 Jan 2014 | 08:32:29 | 08:34:06 | 4 | Y |
| 91 | Fri 31 Jan 2014 | 13:37:56 | 13:41:41 | 3 | Y | Fri 31 Jan 2014 | 09:38:11 | 09:41:11 | 9 | Y |
| 92 | Fri 31 Jan 2014 | 08:47:01 | 08:48:30 | 2 | Y | Fri 31 Jan 2014 | 09:32:56 | 09:36:47 | 10 | Y |
| 93 | Fri 31 Jan 2014 | 08:39:33 | 08:41:01 | 1 | Y | Fri 31 Jan 2014 | 15:59:10 | 16:04:23 | 4 | Y |
| 94 | Fri 31 Jan 2014 | 11:08:22 | 11:10:45 | 3 | Y | Fri 31 Jan 2014 | 11:19:21 | 11:28:32 | 28 | Y |
| 95 | Fri 7 Feb 2014 | 07:50:53 | 07:56:03 | 12 | Y | Fri 7 Feb 2014 | 07:36:20 | 07:36:25 | 1 | Y |
| 96 | Fri 7 Feb 2014 | 08:47:04 | 08:50:54 | 4 | Y | Fri 7 Feb 2014 | 08:53:12 | 08:59:59 | 5 | Y |
| 97 | Fri 7 Feb 2014 | 11:51:44 | 11:53:42 | 2 | Y | Fri 7 Feb 2014 | 07:41:26 | 07:47:02 | 1 | Y |
| 98 | Fri 7 Feb 2014 | 08:13:21 | 08:20:02 | 5 | Y | Fri 7 Feb 2014 | 12:20:16 | 12:22:31 | 5 | Y |
| 99 | Fri 7 Feb 2014 | 09:32:18 | 09:36:49 | 4 | Y | Fri 7 Feb 2014 | 14:50:42 | 14:56:43 | 11 | Y |
| 100 | Fri 7 Feb 2014 | 12:31:55 | 12:36:53 | 12 | Y | Fri 7 Feb 2014 | 15:13:03 | 15:19:48 | 14 | Y |
| 101 | Fri 14 Feb 2014 | 08:15:45 | 08:22:16 | 5 | Y | Fri 14 Feb 2014 | 13:56:55 | 14:00:49 | 6 | Y |
| 102 | Fri 14 Feb 2014 | 09:44:08 | 09:47:44 | 3 | Y | Fri 14 Feb 2014 | 13:56:55 | 14:00:49 | 6 | Y |
| 103 | Fri 14 Feb 2014 | 10:23:43 | 10:24:50 | 2 | Y | Fri 14 Feb 2014 | 09:43:30 | 09:49:29 | 5 | Y |
| 104 | Fri 14 Feb 2014 | 09:42:40 | 09:49:20 | 6 | Y | Fri 14 Feb 2014 | 10:36:00 | 10:39:52 | 17 | Y |
| 105 | Fri 14 Feb 2014 | 09:54:54 | 09:57:44 | 3 | Y | Fri 14 Feb 2014 | 15:38:11 | 15:45:14 | 13 | Y |

**Supplementary Table 4c cont.**

| | | | | | | | | | | |
|---|---|---|---|---|---|---|---|---|---|---|
| 106 | Fri 14 Feb 2014 | 10:08:49 | 10:08:53 | 1 | Y | Fri 14 Feb 2014 | 10:40:23 | 10:46:19 | 7 | Y |
| 107 | Fri 21 Feb 2014 | 09:34:31 | 09:34:41 | 2 | Y | Fri 21 Feb 2014 | 11:34:15 | 11:40:16 | 5 | Y |
| 108 | Fri 21 Feb 2014 | 09:52:18 | 09:57:58 | 3 | Y | Fri 21 Feb 2014 | 07:13:17 | 07:21:21 | 3 | Y |
| 109 | Fri 21 Feb 2014 | 12:16:27 | 12:21:18 | 5 | Y | Fri 21 Feb 2014 | 15:29:38 | 15:33:13 | 5 | Y |
| 110 | Fri 21 Feb 2014 | 13:15:58 | 13:16:41 | 2 | Y | Fri 21 Feb 2014 | 12:36:54 | 12:41:28 | 5 | Y |
| 111 | Fri 28 Feb 2014 | 09:20:34 | 09:25:46 | 9 | Y | Fri 28 Feb 2014 | 11:59:45 | 12:02:19 | 8 | Y |
| 112 | Fri 28 Feb 2014 | 09:20:34 | 09:25:46 | 9 | Y | Fri 28 Feb 2014 | 15:40:09 | 15:43:04 | 8 | Y |
| 113 | Fri 28 Feb 2014 | 09:20:34 | 09:25:46 | 9 | Y | Fri 28 Feb 2014 | 16:35:11 | 16:36:59 | 3 | Y |
| 114 | Fri 28 Feb 2014 | 11:33:23 | 11:36:58 | 10 | Y | | | | | |
| 115 | Fri 28 Feb 2014 | 11:20:09 | 11:27:07 | 7 | Y | Fri 28 Feb 2014 | 14:29:40 | 14:32:34 | 8 | Y |
| 116 | Fri 28 Feb 2014 | 12:01:51 | 12:09:19 | 3 | Y | Fri 28 Feb 2014 | 15:40:09 | 15:43:04 | 8 | Y |

Rel.: consecutive release number, GE: gathering event, Birds: number of birds present at the gathering event, Follow-up: whether birds returned to the feeder after the gathering event (Y: yes, N: no)
